# A novel protein encoded by circINSIG1 reprograms cholesterol metabolism by promoting the ubiquitin-dependent degradation of INSIG1 in colorectal cancer

**DOI:** 10.1186/s12943-023-01773-3

**Published:** 2023-04-22

**Authors:** Li Xiong, Hua-shan Liu, Chi Zhou, Xin Yang, Liang Huang, Hai-qing Jie, Zi-wei Zeng, Xiao-bin Zheng, Wen-xin Li, Zhan-zhen Liu, Liang Kang, Zhen-xing Liang

**Affiliations:** 1 Department of Colorectal Surgery, The Sixth Affiliated Hospital, Sun Yat-sen University, 26 Yuancun Erheng Rd, Guangzhou, 510655 Guangdong China; 2 Department of General Surgery, Guangdong Provincial Key Laboratory of Colorectal and Pelvic Floor Diseases, The Sixth Affiliated Hospital, Sun Yat-sen University, Guangzhou, Guangdong China; 3 State Key Laboratory of Oncology in South China, Collaborative Innovation Center for Cancer Medicine, Sun Yat-sen University Cancer Center, Guangzhou, China; 4 Department of Colorectal Surgery, Sun Yat-sen University Cancer Center, Guangzhou, China

**Keywords:** Hypoxia, Colorectal cancer, Circular RNAs, Cholesterol metabolism

## Abstract

**Background:**

Hypoxia is a hallmark of solid tumors and leads to the metabolic reprogramming of cancer cells. The role of epigenetic regulation between hypoxia and aberrant cholesterol metabolism in colorectal cancer (CRC) remains elusive.

**Methods:**

Hypoxia-responsive circular RNAs (circRNAs) were identified by high throughput RNA sequencing between CRC cells cultured under normoxia or hypoxia. The protein-coding potential of circINSIG1 was identified by polysome profiling and LC–MS. The function of circINSIG1 was validated in vitro and in vivo by gain or loss of function assays. Mechanistic results were concluded by immunoprecipitation analyses.

**Results:**

A novel hypoxia-responsive circRNA named circINSIG1 was identified, which was upregulated in CRC tissues and correlated with advanced clinical stages and poor survival. Mechanistically, circINSIG1 encoded a 121 amino acid protein circINSIG1-121 to promote K48-linked ubiquitination of the critical cholesterol metabolism regulator INSIG1 at lysine 156 and 158 by recruiting CUL5-ASB6 complex, a ubiquitin E3 ligase complex, thereby inducing cholesterol biosynthesis to promote CRC proliferation and metastasis. The orthotopic xenograft tumor models and patient-derived xenograft models further identified the role of circINSIG1 in CRC progression and potential therapeutic target of CRC.

**Conclusions:**

circINSIG1 presents an epigenetic mechanism which provides insights into the crosstalk between hypoxia and cholesterol metabolism, and provides a promising therapeutic target for the treatment of CRC.

**Supplementary Information:**

The online version contains supplementary material available at 10.1186/s12943-023-01773-3.

## Background

Hypoxia is a hallmark of solid tumors and is closely associated with tumor progression [1]. Colorectal cancer (CRC) is a highly lethal cancer with an increasing incidence worldwide [2]. Even with surgical resection and highly aggressive chemotherapies, the mortality rate of CRC still remains high ascribing to recurrence and distant organ metastasis [3]. Tumor hypoxia is associated with poor overall survival, disease-free survival, and loco-regional control [4]. Therefore, further investigation of the molecular mechanisms between CRC and hypoxia is crucial for the advancement of effective therapeutic strategies.

Circular RNAs (circRNAs) are a group of endogenous non-coding RNAs, circularized by a back-splicing reaction [5]. Over the past decade, the key roles of circRNAs in cancer development and progression through diverse mechanisms of action have been identified [6]. Translation is an important mechanism by which circRNAs exert their functions. For example, circ-E-Cad encodes a peptide, C-E-Cad, which promoted glioblastoma tumorigenicity through activation of EGFR-STAT3 signaling pathway [7]. Moreover, studies have reported that some circRNAs encode functional peptides or proteins in CRC. circPPP1R12A and circFNDC3B have been shown to regulate colon cancer progression by encoding novel proteins [8, 9]. In our previous study, we also found that circPLCE1 inhibits CRC progression by encoding a novel NF-κB regulator to promote RPS3 ubiquitin‑dependent degradation [10]. However, the mechanisms of hypoxia-induced circRNAs, especially protein-coding circRNAs, in CRC progression remain largely unknown.

Cholesterol is an essential lipid for multiple cellular physiological processes. Increased cholesterol biosynthesis and uptake, as well as accumulation of cholesterol derivatives, are hallmarks of many cancers [11]. Reprogrammed cholesterol metabolism substantially facilitates cancer progression by regulating cell proliferation, migration and invasion [12]. In addition, hypoxia-inducible factor 1α (HIF-1α), a pivotal hallmark of hypoxia, is highly overexpressed in a variety of cancers and contributes to the aggressive and metastatic phenotype of tumors [13]. The relationship between HIF-1α dependent regulation of fatty acid synthesis and cancer cell progression has been identified [14]. However, the mechanisms between hypoxia and aberrant cholesterol metabolism, especially epigenetic regulation mechanisms, remain unclear in CRC.

In this study, we found that hypoxia-induced circRNA circINSIG1 was upregulated in CRC tissues and was associated with clinical stages and survival. circINSIG1 encoded a 121 amino acid protein to promote the ubiquitin-dependent degradation of the critical cholesterol metabolism regulator INSIG1, thereby enhancing cholesterol biosynthesis to promote CRC proliferation and metastasis. Collectively, our findings reveal hypoxia-associated circINSIG1 as a crucial regulator in cholesterol-driven CRC progression and provide attractive strategies for CRC diagnosis and treatment.

## Methods

Detailed procedures were provided in Supplemental Methods.

### Cell lines and cell cultures

The human CRC cell lines (HCT8, DLD1, HCT116, SW620, CaCO2 and HT29), normal human intestinal epithelial cell lines (HIEC-6) and human embryonic kidney 293T cells were purchased from American Type Culture Collection (ATCC). All of the cells were cultured at 37 °C in Dulbecco’s Modified Eagle Medium (DMEM; Gibco, Thermo Fisher Scientific, St Peters, MO, USA) supplemented with 10% fetal bovine serum (FBS; Gibco, Thermo Fisher Scientific, St Peters, MO, USA) in a 5% CO_2_ atmosphere. As to hypoxic treatment, cells were cultured in a tri-gas incubator with 1% O2, 94% N2 and 5% CO2 or treated with 100 µM CoCl2 (Sigma-Aldrich, MO, USA).

### Animal experiments

For the construction of orthotopic xenograft CRC mouse model, 2 × 10^6^ DLD1 cells transfected with indicated vector were co-injected into the wall of the cecum in 6-week old NOD-SCID mice. Each group consisted of 5 mice. After 8 weeks, all the mice were sacrificed. Intestines and livers were harvested to assess the tumor burden. Cryosections of the harvested organs were stained using H&E for histological assessment. RNA from the rest of organs was extracted for qRT-PCR analysis of human hypoxanthine phosphoribosyltransferase (HPRT) mRNA expression.

For the construction of CRC PDX model, Fresh tumor tissues were obtained from six CRC patients and implanted into NCG mice. When the tumor size reached 1.5 cm [3], the tumors were divided into equal volume ~ 2 mm [3] and were subcutaneously implanted into 4–5 weeks old male NOD-SCID mice. When the tumor size reached about 100 mm [3], all mice were randomized into two groups (n = 5 per group): shNC group and shcircINSIG1 group and treated with intratumor injection of appropriate lentivirus. All mice were sacrificed 4 weeks later and subcutaneous tumors were subjected to H&E, IHC and protein extraction.

### Statistical analysis

GraphPad Prism Software (GraphPad Software, La Jolla, CA, USA) was used to perform statistical analysis. Two-tailed t-Student’s test and one-way ANOVA analysis were performed for statistical comparisons. All statistics analysis data were expressed as mean ± standard error of the mean. A *p* value < 0.05 was considered statistically significant.

## Results

### Characterization and clinical signification of hypoxia-induced circINSIG1 in CRC

**Fig. 1:**
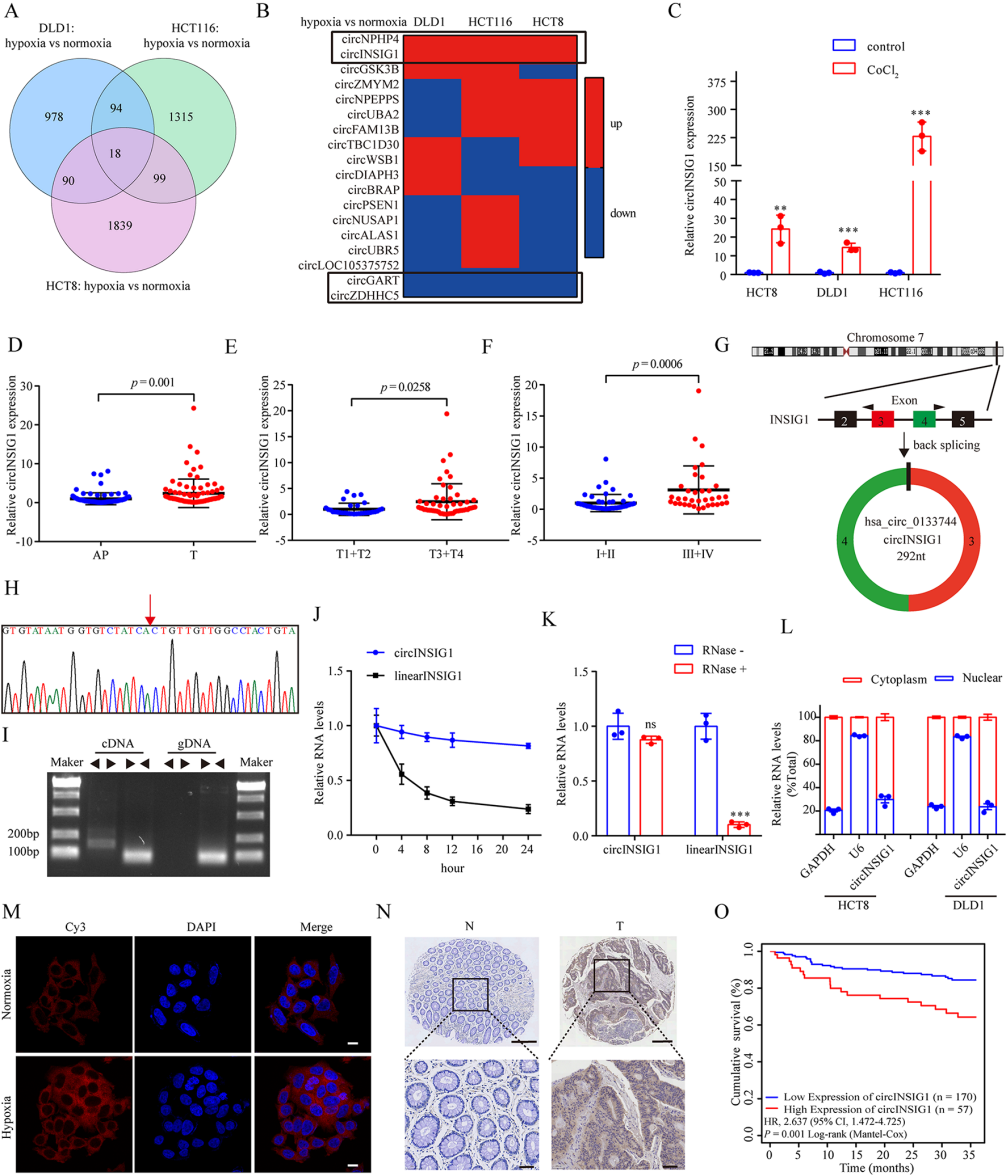
Characterization and clinical signification of hypoxia-induced circINSIG1 in CRC. **A** Differentially expressed circRNAs under hypoxia by circRNAs sequencing in HCT8, HCT116 and DLD1 cells. **B** Heatmap of the expression of 18 circRNAs in HCT8, HCT116 and DLD1 cells under hypoxia. **C** qRT-PCR analysis of circINSIG1 expression in CoCl2-induced cellular pseudo-hypoxia models. **D** qRT-PCR analysis of circINSIG1 expression in 85 paired CRC samples and normal adjacent tissues. **E** Comparison of circINSIG1 expression between patients with T stage 3–4 (n = 53) and those with T stage 1–2 (n = 32), detected by qRT-PCR. **F** Comparison of circINSIG1 expression between patients with clinical stage III–IV (n = 35) and those with clinical stage I–II (n = 50), detected by qRT-PCR. **G** Genomic loci of the INSIG1 gene and circINSIG1. **H** Red arrow indicates the back-splicing of INSIG1 exon 3 and 4 confirmed by Sanger sequencing. **I** RT-PCR analysis of the existence of circINSIG1 with the divergent primers and convergent primers in complementary DNA (cDNA) and genomic DNA (gDNA). **J** qRT-PCR analysis of the abundance of circINSIG1 and INSIG1 linear mRNA in HCT8 cells treated with actinomycin D at the indicated time points. **K** qRT-PCR analysis of circINSIG1 and INSIG1 linear mRNA treated with or without RNase R. **L** qRT-PCR analysis of circINSIG1 location in the nucleus or cytoplasm in HCT8 and DLD1 cells. GADPH served as a marker of cytoplasmic location, while U6 served as a marker of nuclear location. **M** Representative FISH images of circINSIG1 staining in HCT8 cells. Scale bar = 10 μm. **N** Representative images of ISH circINSIG1 expression in the paraffin-embedded CRC tissues and paired normal adjacent tissues. Scale bar = 250 μm and 50 μm respectively. **O** Kaplan-Meier curves for survival of CRC patients with low vs. high expression of circINSIG1. N, normal adjacent tissues; T, tumor tissues. Values are represented as mean ± SD. **p < 0.01, ***p < 0.001; ns, no significance, by 2-tailed Student’s t test

To identify hypoxia-associated circRNAs in CRC, we performed circRNAs sequencing in HCT8, HCT116 and DLD1 cells cultured under normoxia or hypoxia (1% O_2_, 48 h). The results indicated that 18 circRNAs were dysregulated in these three cell lines (Fig. 1A). Then, four circRNAs (circNPHP4, circINSIG1, circGART and circZDHHC5) with the same trend under hypoxia were chosen for further identification (Fig. 1B). The expression levels of these circRNAs were analyzed in CoCl_2_ induced cellular pseudo-hypoxia models and 32 paired CRC samples by quantitative real-time PCR (qRT-PCR), and circINSIG1 (hsa_circ_0133744) was selected for further investigation due to its significant upregulation in CoCl_2_-treated CRC cell lines and CRC tissue samples (Fig. 1C, S1A and S1B). We further analyzed circINSIG1 expression level in 85 paired CRC samples and confirmed that circINSIG1 was upregulated in CRC (Fig. 1D). Further analysis revealed that circINSIG1 expression was higher in patients with advanced T stages or clinical stages (Fig. 1E F).

circINSIG1 was formed by the back-splicing of exon 3 and 4 of the insulin induced gene 1 (INSIG1) with 292nt (Fig. 1G). The back-splicing junction of circINSIG1 was verified by Sanger sequencing (Fig. 1H). Divergent primers and convergent primers were used to detect circINSIG1 and linear transcripts of INSIG1 in both complementary DNA (cDNA) and genomic DNA (gDNA). The results indicated that circINSIG1 could only be amplified in cDNA by the divergent primers (Fig. 1I). Half-life assays showed that circINSIG1 was much more stable than linear INSIG1 mRNA (Fig. 1J). Moreover, circINSIG1 was observed to resist RNase R digestion (Fig. 1K). Nuclear mass separation assays and fluorescence in situ hybridization (FISH) assays demonstrated the enrichment of circINSIG1 in the cytoplasm of CRC cells (Fig. 1L M).

In addition, we analyzed circINSIG1 expression in a large cohort of CRC patients by in situ hybridization (ISH; n = 227, Table S1). The results also confirmed that the expression level of circINSIG1 was increased in CRC tissues compared with paired normal adjacent tissues (Fig. 1N). The expression of circINSIG1 in the cohort was significantly correlated with clinicopathological characteristics of CRC patients, such as TNM stages and clinical stages (p < 0.05, Table S1). Moreover, Kaplan-Meier curves showed that higher circINSIG1 expression was associated with poorer survival in CRC patients (Fig. 1O). Taken together, these data demonstrated the circularity and clinical significance of circINSIG1.

### circINSIG1 encodes a 121 amino acid protein

**Fig. 2:**
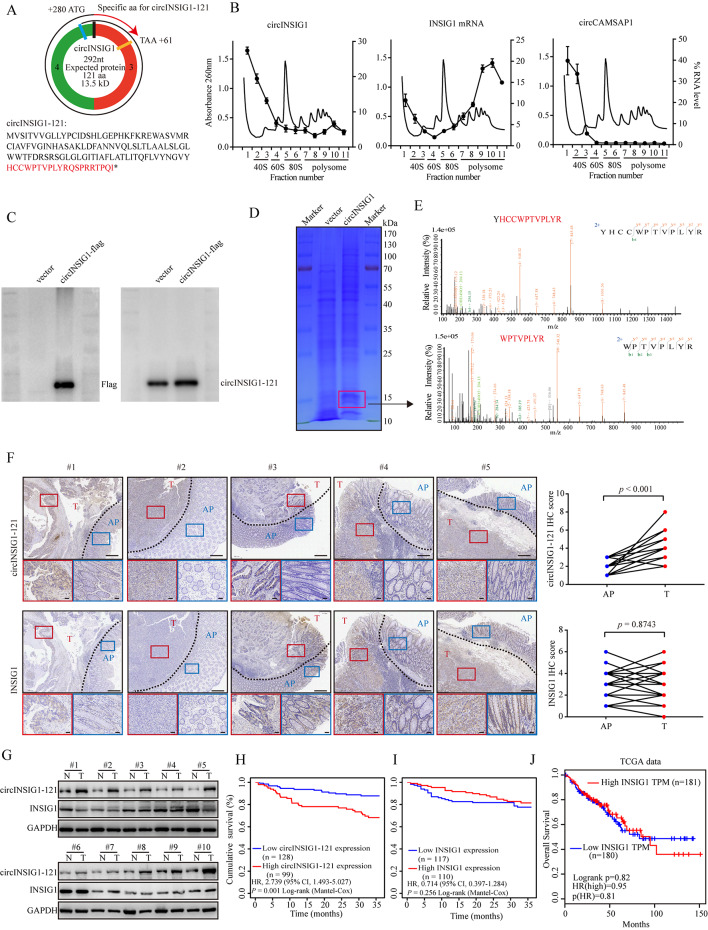
circINSIG1 encodes a 121 amino acid protein. **A** Upper panel, the putative open reading frame (ORF) in circINSIG1. Lower panel, the sequences of the putative ORF encoded amino acid sequences are shown. **B** Polysome profiling in cytoplasmic extracts of HCT8 cells were fractionated through sucrose gradients, and the relative expression of circINSIG1, INSIG1 mRNA and circCAMSAP1 were analyzed by qRT-PCR in the gradient fractions. INSIG1 mRNA served as a positive control. circCAMSAP1 served as a negative control. **C** Western blot analysis of the expression of circINSIG1-121 with Flag antibody and circINSIG1-121 antibody. **D** The lysates from HCT8 cells with circINSIG1 overexpression were separated by SDS-PAGE. Protein bands near 14 KD were excised manually and summited for identification by LC–MS/MS. **E** The identified circINSIG1-121 unique peptide sequences. **F** Immunohistochemistry (IHC) images of circINSIG1-121 and INSIG1 expression in CRC tissues (left) and semiquantitative scoring of circINSIG1-121 and INSIG1 expression in 20 CRC tissues (right). **G** Western blot analysis of circINSIG1-121 expression in 10 paired CRC samples and normal adjacent tissues. **H** Kaplan-Meier curves for survival of CRC patients with low vs. high expression of circINSIG1-121. **I** Kaplan-Meier curves for survival of CRC patients with low vs. high expression of INSIG1. **J** Kaplan-Meier curves for survival of CRC patients with INSIG1 levels in the TCGA database. N, normal adjacent tissues; T, tumor tissues. Values are represented as mean ± SD, by 2-tailed Student’s t test

Accumulating data suggest that some circRNAs could encode functional peptides [7, 15]. To determine the protein-coding potential of circINSIG1, we first analyzed the putative open reading frame (ORF) and internal ribosome entry site (IRES) of circINSIG1 as annotated in the database circRNADb. The results showed that a spanning junction ORF with the potential to encode a 121 amino acid protein (referred as circINSIG1-121 hereafter) and an IRES at 207–292 nt were contained in the sequence of circINSIG1 (Fig. 2A). Next, sucrose density gradient centrifugation based polysome profiling was performed. Ribosomes in the cell lysate were divided into 40 and 60 S ribosomal subunits, as well as monosomes (80 S) and polysomes (Fig. 2B). Linear INSIG1 mRNA served as a positive control and circCAMSAP1, a circRNA has been identified without protein encoding ability in our previous study [16], served as a negative control. Notably, the results indicated that circINSIG1 could be detected in the monosomes and polysomes fractions (Fig. 2B). Additionally, the activity of the IRES driving the translation of the ORF was validated by dual-luciferase assays (Figure S2A and S2B). Due to the lack of a stop codon in the first-round read, circINSIG1-121 had a unique 20 amino acid tail at its C-terminus formed by a natural frameshift in the second-round translation (Fig. 2A). A monoclonal antibody against the tail 20 amino acid sequences was produced by NovoPro Bioscience to detect circINSIG1-121 expression. Then, a circINSIG1-flag vector was constructed and transfected into CRC cells. Flag antibody detected a ~ 14KD band in cells transfected with circINSIG1-flag vector, but not in cells transfected with control vector (Fig. 2C, left). Furthermore, the endogenous existence of circINSIG1-121 was confirmed by circINSIG1-121 antibody (Fig. 2C, right). Mass spectrometry followed by SDS-PAGE using circINSIG1 overexpressing CRC cells further verified the specific peptide fragments of circINSIG1-121 protein with predicted molecular weight (Fig. 2D and E and S2C).

Next, the protein levels of circINSIG1-121 and INSIG1 were analyzed in 20 and 10 paired CRC samples by immunohistochemistry (IHC) and western blot respectively. The results revealed that circINSIG1-121 was upregulated in CRC tissues, whereas the protein level of INSIG1 showed no significant difference between paired normal adjacent tissues and CRC tissues (Fig. 2F and G). Moreover, the protein level of circINSIG1-121 and INSIG1 were examined in a CRC cohort by IHC (n = 227). The results also indicated that higher circINSIG1-121 expression was associated with poorer survival in CRC patients (Fig. 2H). In contrast, the linear INSIG1 expression levels did not have a correlation with the survival of CRC patients in this study cohort or in the TCGA database (Fig. 2I J). Altogether, these data confirm that circINSIG1 encodes a 121 amino acid novel protein, which is correlated with poor prognosis in CRC.

### circINSIG1-121 promotes CRC proliferation and metastasis

**Fig. 3:**
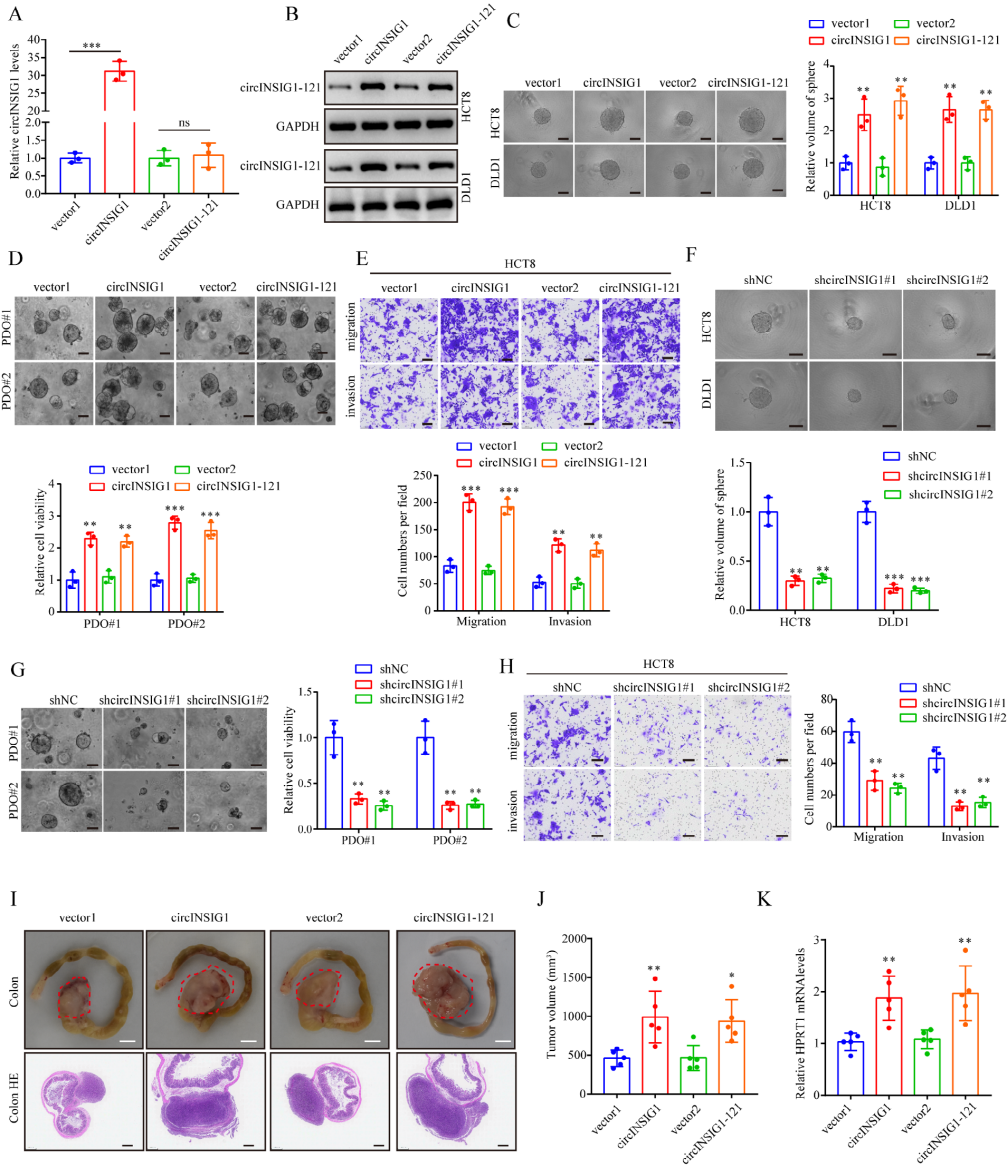
circINSIG1-121 promotes CRC proliferation and metastasis. **A** qRT-PCR analysis of circINSIG1 expression in HCT8 cells with circINSIG1 or circINSIG1-121 overexpression. **B** Western blot analysis of circINSIG1-121 expression in HCT8 and DLD1 cells with circINSIG1 or circINSIG1-121 overexpression. **C** Sphere formation assays of circINSIG1 or circINSIG1-121 transfected HCT8 and DLD1 cells, n = 3. Scale bar = 200 μm. **D** Patient-derived organoids (PDOs) growth with circINSIG1 or circINSIG1-121 transfection, n = 3. Scale bar = 50 μm. **E** Migration and invasion assays of HCT8 cells with circINSIG1 or circINSIG1-121 overexpression, n = 3. Scale bar = 100 μm. **F** Sphere formation assays of HCT8 and DLD1 cells circINSIG1 knockdown, n = 3. Scale bar = 200 μm. **G** PDOs growth with circINSIG1 knockdown, n = 3. Scale bar = 50 μm. **H** Migration and invasion assays of HCT8 cells with circINSIG1 knockdown, n = 3. Scale bar = 100 μm. **I** Representative images of gross inspection and H&E staining of the CRC orthotopic tumors (n = 5 per group). Orthotopic tumors scale bars = 5 mm, colon H&E scale bars = 500 μm. **J** Statistical analysis of orthotopic tumor volumes. **K** qRT-PCR analysis of human HPRT mRNA relative to mouse 18 S rRNA in the livers of orthotopic xenograft CRC models. Values are represented as mean ± SD. *p < 0.05, **p < 0.01, ***p < 0.001, by one-way ANOVA

To elucidate the biological functions of circINSIG1 in CRC, we first analyzed the expression of circINSIG1 in normal human intestinal epithelial cell lines and CRC cell lines using qRT-PCR. Consistently, circINSIG1 was found to be upregulated in CRC cells (Figure S3A). Moreover, HCT8 and DLD1 CRC cell lines were selected to conduct cell experiments because of the moderate expression of circINSIG1 (Figure S3A). Then, CRC cell lines with stable overexpression of circINSIG1 or circINSIG1-121 were generated (Fig. 3A and B and S3B). Sphere formation assays identified that circINSIG1 or circINSIG1-121 enhanced CRC cell proliferation (Fig. 3C). Besides, we overexpressed circINSIG1 or circINSIG1-121 in CRC patient-derived organoids (PDOs) and the results also indicated that circINSIG1 or circINSIG1-121 facilitated the growth of PDOs (Fig. 3D). Likewise, overexpression of circINSIG1 or circINSIG1-121 promoted CRC cell migration and invasion (Fig. 3E and S3C). Moreover, we knocked down circINSIG1 by two short hairpin RNAs (shRNAs) specifically targeting the back-spliced junction of circINSIG1 in HCT8 and DLD1 cells (Figure S3D and S3E). As expected, knockdown of circINSIG1 or circINSIG1-121 significantly inhibited CRC cell growth, migration and invasion (Fig. 3F H and S3F). Additionally, a mutated circINSIG1 vector with the start codon (circINSIG1-ATGmut) was constructed. Overexpression of circINSIG1-ATGmut failed to enhance CRC cell proliferation, migration and invasion (Figure S3G-S3I), suggesting that circINSIG1 promoted CRC cell proliferation and metastasis by encoding circINSIG1-121 instead of the circular RNA form of circINSIG1.

Next, the in vivo function of circINSIG1-121 in CRC progression was determined using orthotopic xenograft tumor models. The results verified that overexpression of circINSIG1 or circINSIG1-121 significantly promoted tumor growth (Fig. 3I J). In addition, analysis of human HPRT mRNA expression in the mouse livers confirmed that circINSIG1 or circINSIG1-121 could lead to a significant increase in the tumor burden of liver metastasis (Fig. 3K). Collectively, these results demonstrated the critical role of circINSIG1-121 in promoting CRC proliferation and metastasis.

### circINSIG1-121 interacts with INSIG1 to promote ubiquitin-dependent degradation of INSIG1 to induce cholesterol biosynthesis

**Fig. 4:**
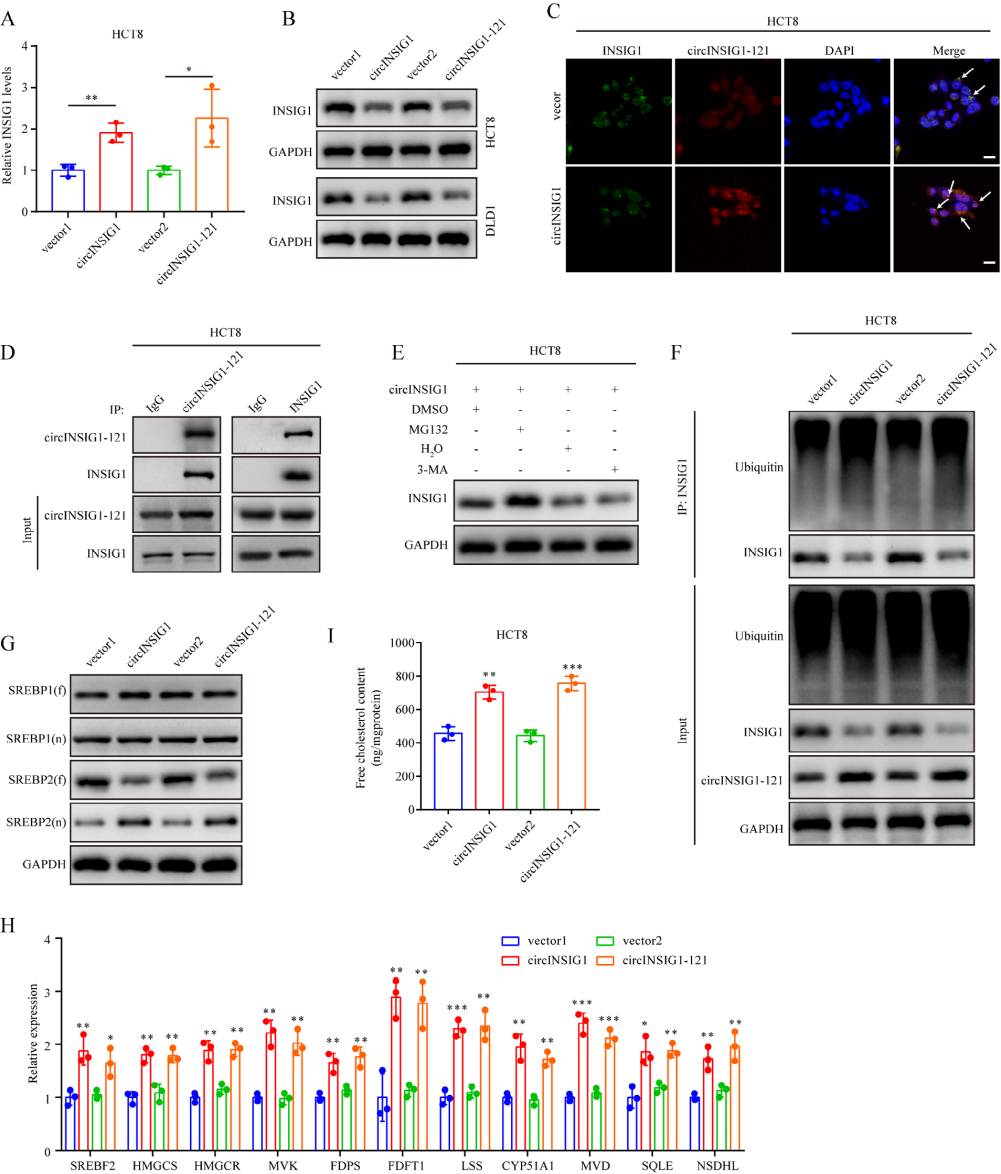
circINSIG1-121 interacts with INSIG1 to promote ubiquitin-dependent degradation of INSIG1 to induce cholesterol biosynthesis. **A** qRT-PCR analysis of INSIG1 expression in HCT8 cells with circINSIG1 or circINSIG1-121 overexpression. **B** Western blot analysis of INSIG1 expression in HCT8 and DLD1 cells with circINSIG1 or circINSIG1-121 overexpression. **C** Immunofluorescence assay showed the colocalization of circINSIG1-121 and INSIG1 in HCT8 cells. Scale bar = 10 μm. **D** Immunoprecipitation assays using circINSIG1-121 or INSIG1 antibodies confirmed the interaction between circINSIG1-121 and INSIG1 in HCT8 cells. **E** Western blot analysis of INSIG1 expression in HCT8 transfected with circINSIG1 and treated with MG132 (10 µM) or 3-Methyladenine (3-MA) (10 mg/ml). **F** Western blot analysis of INSIG1 ubiquitin levels after immunoprecipitation using INSIG1 antibody in HCT8 cells transfected with circINSIG1 or circINSIG1-121 vector. **G** Western blot analysis of full-length SREBPs (fSREBPs) and active nuclear SREBPs (nSREBPs) protein levels in HCT8 cells transfected with the indicated plasmids. **H** qRT-PCR analysis of SREBP2 target genes in HCT8 cells with circINSIG1 or circINSIG1-121 overexpression. **I** Free cholesterol content detection in HCT8 cells with circINSIG1 or circINSIG1-121 overexpression. Values are represented as mean ± SD. *p < 0.05, **p < 0.01, ***p < 0.001, by one-way ANOVA

To identify the molecular mechanism by which circINSIG1-121 promotes CRC progression, we analyzed the RNA and protein levels of INSIG1 in HCT8 and DLD1 cells with circINSIG1 or circINSIG1-121 overexpression. Interestingly, the RNA levels of INSIG1 were increased with circINSIG1 or circINSIG1-121 overexpression, whereas the protein levels of INSIG1 were decreased (Fig. 4A and B and S4A). Moreover, we observed the colocalization of circINSIG1-121 and INSIG1 in CRC cells by immunofluorescence (Fig. 4C). Immunoprecipitation (IP) assays confirmed that circINSIG1-121 interacted with INSIG1 (Fig. 4D).

It has been reported that phosphorylation of INSIG1 promotes the degradation of INSIG1 protein [17]. Therefore, we first analyzed the phosphorylation level of INSIG1 and found that the expression of phosphorylated INSIG1 (p-INSIG1) was not increased with circINSIG1 or circINSIG1-121 overexpression (Figure S4B). These results implied that circINSIG1 or circINSIG1-121 may not regulate the degradation of INSIG1 by affecting its phosphorylation level. Since the ubiquitin-proteasome and autophagy-lysosome pathways are two major systems for protein clearance in eukaryotic cells [18], we treated CRC cells with the proteasome inhibitor MG132 or autophagic-sequestration inhibitor 3-methyladenine (3-MA) to further investigate the molecular mechanisms underlying circINSIG1-121-mediated INSIG1 degradation. We observed that MG132, but not 3-MA, enhanced the protein level of INSIG1, and the ubiquitylation level of INSIG1 was increased in CRC cells with circINSIG1 or circINSIG1-121 overexpression, indicating that circINSIG1-121 facilitated INSIG1 degradation through the ubiquitin-proteasome pathway (Fig. 4E F).

Sterol regulatory element–binding proteins (SREBPs) play a vital role in cholesterol and lipid homeostasis in mammals [19]. INSIG proteins bind to SREBPs and SREBP-cleavage-activating protein (SCAP) complex to anchor this complex in the endoplasmic reticulum (ER) to regulate lipogenesis [20]. Besides, SREBP1 is responsible for fatty acid synthesis and energy storage, while SREBP2 is responsible for cholesterol regulation [21]. Therefore, we first analyzed the protein levels of full-length SREBPs (fSREBPs) and active nuclear SREBPs (nSREBPs). Western blot analysis revealed a marked increase in nSREBP2, but no significant change in nSREBP1 in CRC cells overexpressing circINSIG1 or circINSIG1-121, suggesting that circINSIG1-121 might promote SREBP2 activation to enhance cholesterol biosynthesis (Fig. 4G). Moreover, the target genes of SREBP1 and SREBP2 were analyzed, and the results indicated that the expression levels of SREBP2 target genes were increased, while the expression of SREBP1 target genes did not altered significantly (Fig. 4H and S4C). In agreement with the augmented expression of cholesterol biosynthetic genes, free cholesterol content was increased with circINSIG1-121 overexpression compared to the control group (Fig. 4I). Taken together, these data suggest that circINSIG1-121 promotes ubiquitin-dependent degradation of INSIG1, leading to the release of SREBP2-SCAP complex from the ER, which subsequently induces cholesterol biosynthesis.

To confirm whether circINSIG1 reprograms cholesterol in an INSIG1 dependent manner, in vitro rescue assays were performed. As shown in the Figure S S4D-F, overexpressing circINSIG1 promoted CRC cell proliferation, migration and invasion, which could be significantly antagonized by overexpression of INSIG1. Moreover, overexpressing circINSIG1 increased the expression levels of SREBP2 target genes and the content of free cholesterol in CRC cells, which could be abolished by overexpression of INSIG1 (Figure S4G and H). These data suggest that circINSIG1 reprograms cholesterol metabolism and facilitates malignant progress in CRC, at least in part, by regulating INSIG1 levels.

### circINSIG1-121 recruits CUL5-ASB6 complex to promote K48-linked ubiquitination of INSIG1

**Fig. 5:**
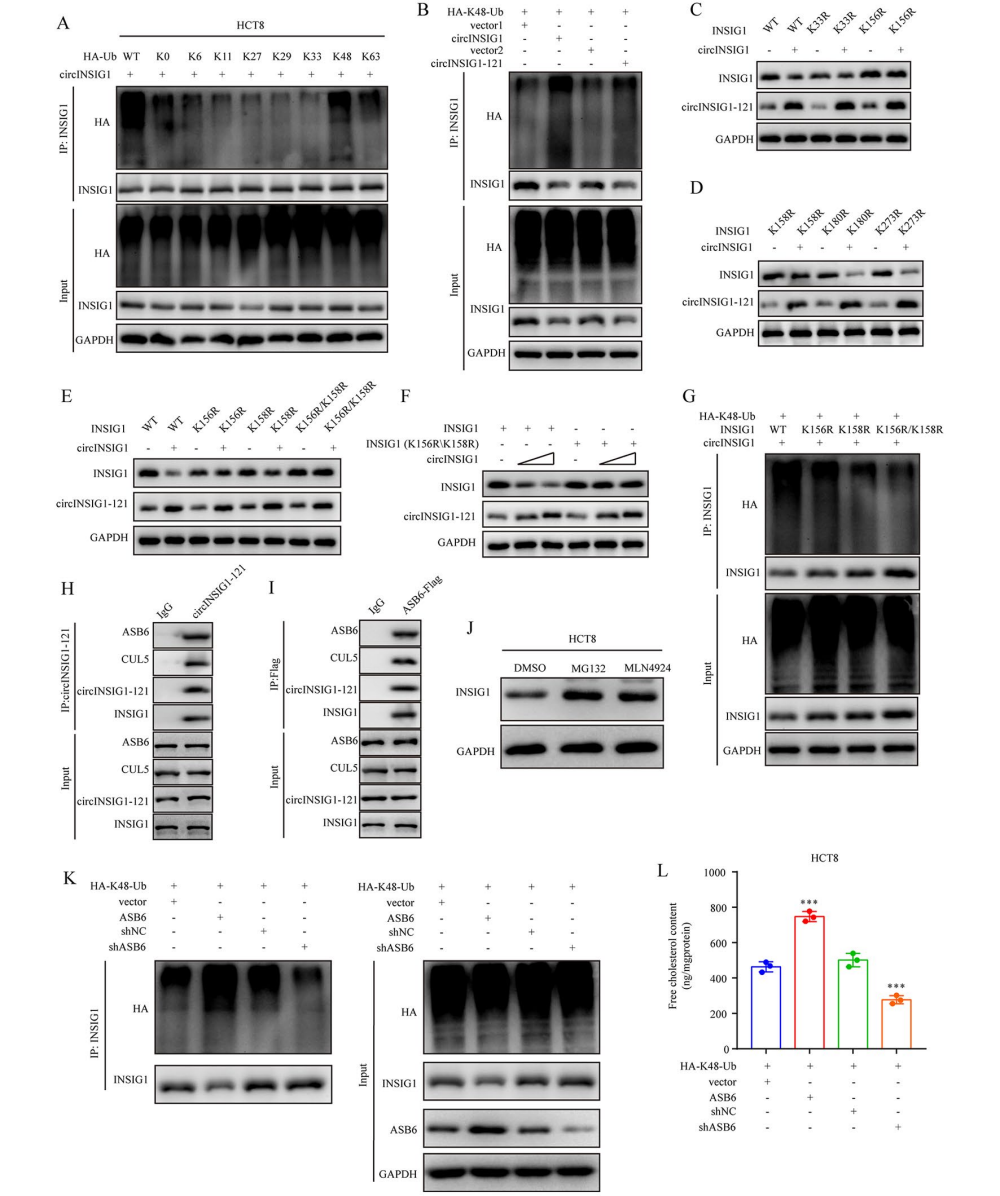
circINSIG1-121 recruits CUL5-ASB6 complex to promote K48-linked ubiquitination of INSIG1. **A** Western blot analysis of INSIG1 ubiquitin levels after immunoprecipitation using INSIG1 antibody in HCT8 cells transfected with HA-tagged wildtype (WT) Ub or different ubiquitin chain vectors (K0, K6, K11, K27, K29, K33, K48, K63). **B** Western blot analysis of K48-linked ubiquitin levels of INSIG1 after immunoprecipitation using INSIG1 antibody in HCT8 cells transfected with circINSIG1 or circINSIG1-121 along with HA-K48-Ub vector. **C** and **D** Western blot analysis of INSIG1 and circINSIG1-121 expression in HCT8 cells transfected with circINSIG1 and indicated mutants. **E** Western blot analysis of INSIG1 and circINSIG1-121 expression in HCT8 cells transfected with circINSIG1 and indicated mutants. **F** Western blot analysis of INSIG1 and circINSIG1-121 expression in HCT8 cells transfected with circINSIG1 and indicated mutants. **G** Western blot analysis of K48-linked ubiquitin levels of INSIG1 after immunoprecipitation using INSIG1 antibody in HCT8 cells transfected with circINSIG1 and indicated mutants along with HA-K48-Ub vector. **H** Western blot analysis of the samples immunoprecipitated by circINSIG1-121 antibody in HCT8 cells. **I** Immunoprecipitation assay was performed in ASB6-Flag transfected HCT8 cells using Flag antibody, followed by Western blot analysis. **J** Western blot analysis of INSIG1 expression in HCT8 cells treated with MG132 (10 µM) or MLN4924 (10 µM). **K** Western blot analysis of K48-linked ubiquitin levels of INSIG1 after immunoprecipitation using INSIG1 antibody in HCT8 cells with ASB6 overexpression or knockdown. **L** Free cholesterol content detection in HCT8 cells with ASB6 overexpression or knockdown. Values are represented as mean ± SD. ***p < 0.001, by one-way ANOVA

Ubiquitin chains serve as a major degradation signal responsible for targeting different types of substrates and different kinds of ubiquitin chains have different biological functions [22]. To identify the kind of ubiquitin chain that leads to INSIG1 degradation, we transfected CRC cells with different HA-labeled ubiquitin chain vectors. Our results revealed that INSIG1 was mainly ubiquitinated with K48 linkage, but not with other types of ubiquitin chains (Fig. 5A). K48-linked polyubiquitination was enhanced in cells with circINSIG1-121 overexpression (Fig. 5B). These findings indicated that circINSIG1-121 mediated the degradation of INSIG1 through K48-linked ubiquitination. Then, we sought to determine which lysine residues of INSIG1 are the major ubiquitination sites that result in INSIG1 degradation. Five conservative lysine (K) points were identified in INSIG1 protein (Figure S5A). Thus, five INSIG1 mutants in which the corresponding lysine residues were individually mutated to arginine (R) were used to transfected CRC cells, respectively. We found that the protein level of INSIG1 in INSIG1 K156R and K158R transfection group was decreased slightly compared to the wildtype group (Fig. 5C and D). To further confirm these 2 ubiquitination sites of INSIG1, INSIG1 mutants with both lysine points mutation was constructed. Subsequent western blot and immunoprecipitation assays demonstrated that the degradation of INSIG1 mediated by circINSIG1-121 was markedly attenuated by this mutant and the K48-linked ubiquitination level of INSIG1 was significantly lower than the wildtype group (Fig. 5E and G). These results illustrate that circINSIG1-121 promotes the K48-linked ubiquitination of INSIG1 at residues K156 and K158.

As circINSIG1-121 is not an E3 ligase, we reasoned that circINSIG1-121 might function as an adaptor to recruit certain E3 ligase to ubiquitinate INSIG1. Immunoprecipitation assays with circINSIG1-121 antibody were performed and mass spectrometry analysis identified the present of ASB6 (Figure S5B). ASB6, a substrate recognition adaptor, can interact with Cullin5 (CUL5) to form a CUL5-ASB6 complex which functions as a ubiquitin E3 ligase complex [23, 24]. Further immunoprecipitation assays validated the interaction between circINSIG1-121 and CUL5-ASB6 complex (Fig. 5H and I). We further found that proteasome inhibitor MG132 and Cullin-Ring E3 ligase activity inhibitor MLN4924 caused an obvious elevation of endogenous INSIG1 protein levels (Fig. 5J). Furthermore, ASB6 overexpression enhanced the K48-linked ubiquitination of INSIG1 and decreased the abundance of INSIG1 protein (Fig. 5K). Conversely, ASB6 knockdown increased the abundance of INSIG1 protein and inhibited K48-linked ubiquitination of INSIG1 (Fig. 5K). In addition, overexpression of ASB6 promoted cholesterol biosynthesis, and vice versa (Fig. 5L). Therefore, circINSIG1-121 recruits CUL5-ASB6 complex to promote K48-linked ubiquitination of INSIG1 at K156 and K158.

### Hypoxia-induced EIF4A3 promotes circINSIG1 expression

**Fig. 6:**
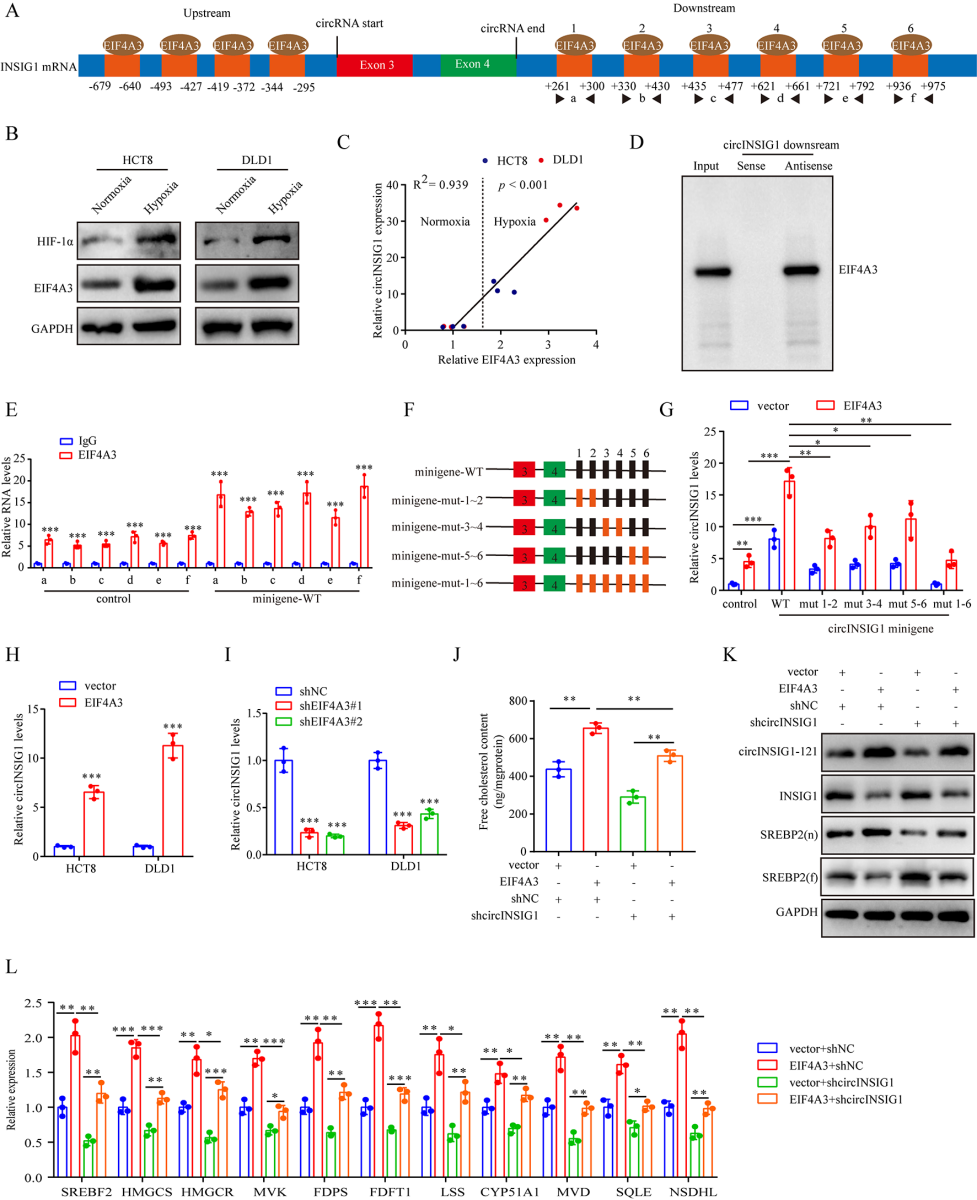
Hypoxia-induced EIF4A3 promotes circINSIG1 expression. **A** The EIF4A3 binding sites on the upstream and downstream of the back-splicing site of circINSIG1 predicted by the CircInteractome. **B** Western blot analysis of EIF4A3 expression in HCT8 and DLD1 cells under hypoxia. **C** The correlation between EIF4A3 and circINSIG1 expression in HCT8 and DLD1 under hypoxia. **D** Western blot analysis of RNA pull-down assays with EIF4A3 antibody. **E** RIP-qPCR assay in HCT8 cells transfected with or without wild-type (WT) circINSIG1 minigene using the indicated primers. IgG served as a control. **F** Schematic of circINSIG1 minigenes with WT or mutant (mut) EIF4A3 binding sites on the flanked intron regions of INSIG1. **G** HCT8 cells with or without EIF4A3 overexpression were transfected with WT or various mutant circINSIG1 minigenes. qRT-PCR analyzed the expression level of circINSIG1 in HCT8 cells. **H** qRT-PCR analysis of circINSIG1 expression in HCT8 and DLD1 cells with EIF4A3 overexpression. **I** qRT-PCR analysis of circINSIG1 expression in HCT8 and DLD1 cells with EIF4A3 knockdown. **J-l** HCT8 cells with or without EIF4A3 overexpression were transfected with shRNA targeting circINSIG1 (shcircINSIG1) for rescue assays. Free cholesterol content detection was detected. (**J**) Western blot analysis of indicated protein expression. (**K**) qRT-PCR analysis of SREBP2 target genes. (**L**) Values are represented as mean ± SD. *p < 0.05, **p < 0.01, ***p < 0.001, by one-way ANOVA

Next, we probed the mechanism of circINSIG1 upregulation in CRC cells. Studies have shown that RNA-binding proteins (RBPs) participate in circRNAs biogenesis by binding to the flanked intron regions [25, 26]. Here, CircInteractome, a tool for exploring circRNAs and their interacting proteins, was used to search for the potential RBPs responsible for the circularization of circINSIG1. Four and six EIF4A3 binding sites were identified upstream and downstream of the back-splicing site of circINSIG1, respectively (Fig. 6A and S6A). EIF4A3 is a core component of the exon junction complex and play an essential role in pre-mRNA splicing [27]. Then, we analyzed EIF4A3 expression in TCGA database and found that the expression of EIF4A3 was upregulated in CRC (Figure S6B). Moreover, EIF4A3 expression was positively correlated with HIF-1α expression, a marker of hypoxia (Figure S6C). Western blot and qRT-PCR assays indicated that the protein expression of EIF4A3 was upregulated under hypoxia, and the expression of circINSIG1 was positively correlated with EIF4A3 expression (Fig. 6B C).

To verify the ability of EIF4A3 to bind to circINSIG1 flanking sequences, RNA pull-down assays were performed. The results showed that EIF4A3 could bind to the downstream flanking sequence of circINSIG1, but not upstream sequences (Fig. 6D and S6D). Then, we constructed a circINSIG1 minigene and performed RNA immunoprecipitation (RIP) assays. As shown in Fig. 6E, the enrichment of the six putative EIF4A3-binding sites was higher in the anti-EIF4A3 group than in the anti-IgG group, while the transfection of circINSIG1 minigene further improved the enrichment, indicating that EIF4A3 could bind to these putative binding sites on the flanked regions. We further constructed CRC cell lines stably overexpressing EIF4A3 and a series of circINSIG1 minigenes with EIF4A3-binding site mutations (Figure S6E and 6 F). Notably, mutation of the putative binding sites individually had little effect on the expression of circINSIG1, while mutation of all six EIF4A3-binding sites substantially reduced circINSIG1 formation (Fig. 6G). These results suggested that these six EIF4A3-binding sites on the downstream flanked regions of circINSIG1 were necessary for EIF4A3-mediated circINSIG1 circularization.

Moreover, we observed that circINSIG1 levels were elevated with EIF4A3 overexpression, whereas decreased by knockdown of EIF4A3 (Fig. 6H and I and S6F). Additionally, overexpression of EIF4A3 promoted SREBP2 target genes activation and cholesterol biosynthesis, which could be partly attenuated by silencing circINSIG1 (Fig. 6J L). Altogether, these data indicate that hypoxia-induced EIF4A3 promotes the biogenesis of circINSIG1.

### circINSIG1 is a potential therapeutic target for CRC patients

**Fig. 7:**
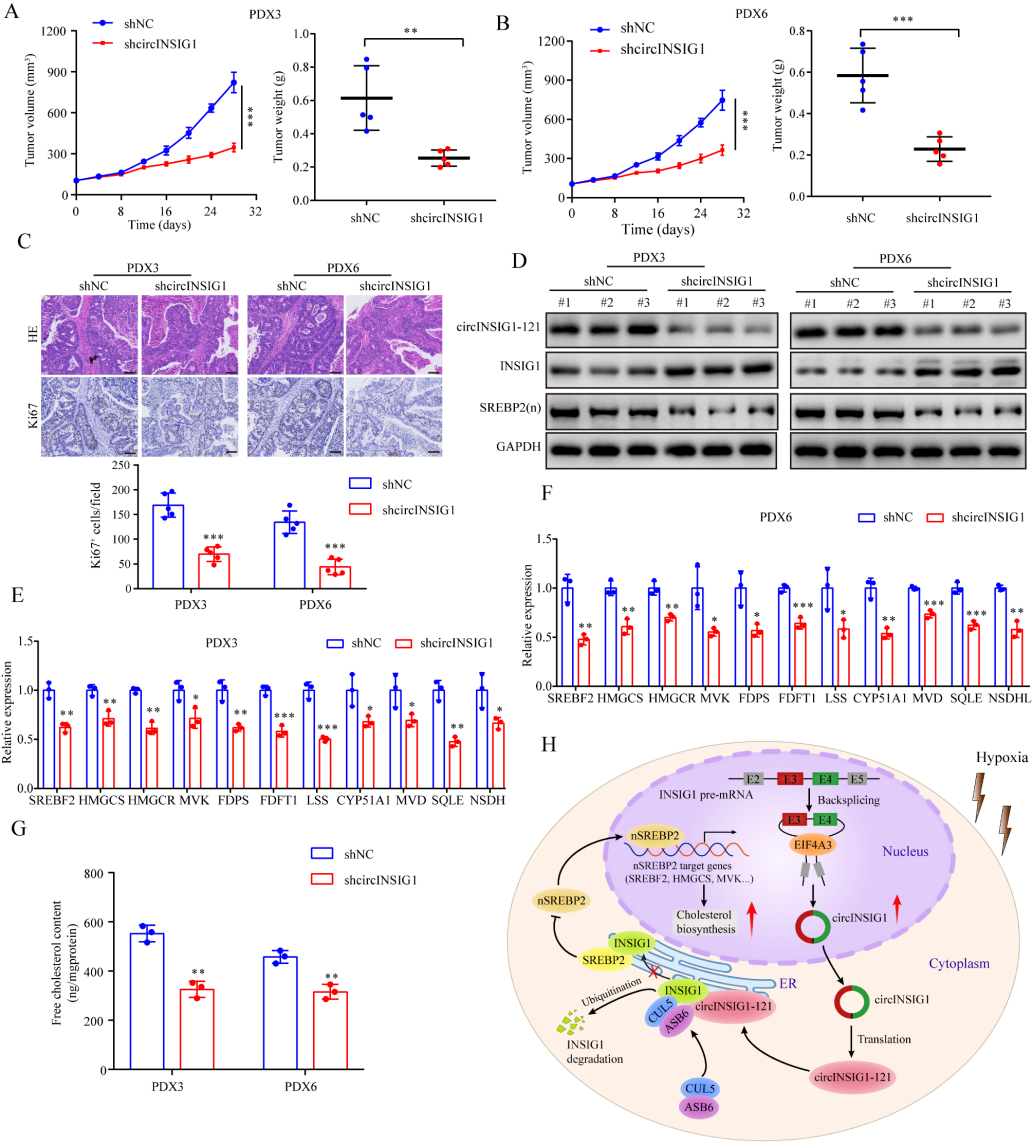
circINSIG1 is a potential therapeutic target for CRC patients. **A** and **B** Growth curves and tumor weight of patient-derived xenograft (PDX) model tumors after treatment with shcircINSIG1 lentivirus (n = 5 per group). **C** H&E and Ki67 staining in PDX model tumors. Scale bar = 100 μm. **D** Western blot analysis of circINSIG1-121, INSIG1 and SREBP2(n) expression in the PDX tumors. GAPDH served as loading control. **E and F** qRT-PCR analysis of SREBP2 target genes expression in the PDX tumors. **G** Free cholesterol content detection in the PDX tumors. **H** A schematic model of circINSIG1 functions in tumor progression. Values are represented as mean ± SD. *p < 0.05, **p < 0.01, ***p < 0.001, by 2-tailed Student’s t test (**A, B** and **G**) and one-way ANOVA (**E** and **F**)

To verify whether circINSIG1 can serve as a potential therapeutic target for CRC, we analyzed the expression of circINSIG1 and circINSIG1-121 in six patient-derived xenograft (PDX) models. Next, we chose PDX3 and PDX6 with circINSIG1 upregulation to examine the therapeutic effect by targeting circINSIG1 (Figure S7A and S7B). The results showed that treatment with shcircINSIG1 lentivirus led to significantly slower tumor growths than the control group (Fig. 7A and B). IHC analysis revealed that the percentage of Ki67-positive cells was significantly lower in shcircINSIG1 lentivirus treatment group (Fig. 7C). To further validate the mechanism of circINSIG1 in vivo, we evaluated the expression of the downstream targets of circINSIG1 and detected cholesterol content in these tumors. The results indicated that knockdown of circINSIG1 decreased the protein level of circINSIG1-121 and suppressed the INSIG1-mediated SREBP1 signaling (Fig. 7D F). Besides, free cholesterol content was reduced in circINSIG1 knockdown PDX tumors (Fig. 7G). Taken together, these data suggest that circINSIG1 is a novel therapeutic target for CRC patients.

## Discussion

Oxygen is essential for energy metabolism to drive cellular bioenergetics. The rapid proliferation of tumors outgrows their surrounding vasculature, resulting in a drop of normal oxygen levels to hypoxic levels [13]. Given the rapid developments in RNA-seq technologies and bioinformatic analyses, the key role of hypoxia-responsive circRNAs in the malignant progression, distant metastasis and recurrence was discovered [28]. However, the function of hypoxia-responsive circRNAs, especially the relationship between circRNAs and hypoxia-induced metabolic reprogramming, still remains elusive in CRC. In this study, we identified a novel hypoxia-responsive circRNA generated from INSIG1 gene, termed as circINSIG1, which was shown to be modulated by hypoxia-induced EIF4A3. Moreover, circINSIG1 encoded a 121 amino acid protein circINSIG1-121 to promote K48-linked ubiquitination of the critical cholesterol metabolism regulator INSIG1 at lysine 156 and 158 by recruiting CUL5-ASB6 complex, thus inducing cholesterol biosynthesis to facilitate CRC proliferation and metastasis. Importantly, circINSIG1 presents an epigenetic mechanism which provides insights into the crosstalk between hypoxia and cholesterol metabolism, and provides a promising therapeutic target for the treatment of CRC.

To date, only two hypoxia-related circRNAs have been reported in hypoxic tumor aggression and metastasis of CRC. For example, hypoxia-responsive circ-Erbin acts as a sponge of miR-125a-5p and miR-138-5p to facilitate the aggression and metastasis of CRC by accelerating the cap-independent protein translation of HIF-1α [29]. Another circRNA named circ-133, derived from hypoxic CRC exosomes, promotes the metastasis of CRC via the miR-133a/GEF-H1/RhoA axis [30]. Both circRNAs exert their functions by acting as miRNA sponges. In the present study, we identified a novel hypoxia-induced circRNA circINSIG1, which encoded a 121 amino acid protein circINSIG1-121 to promote CRC progression.

Cancer cell metabolism is reprogrammed in hypoxia, thus taking advantage from these adaptions to fuel survival, proliferation, and ensure tumor progression. The regulation of the metabolism of glucose, fatty acid and amino acids in hypoxic cancer cells has been reported [31]. Recently, the mechanisms of hypoxia-responsive circRNAs in the metabolic reprogramming of hypoxic tumors have been identified. However, most of the hypoxia-responsive circRNAs functioned by mediating glycolysis, such as circRNF20 in breast cancer [32], circSLAMF6 in gastric cancer [33] and circ_0008450 in hepatocellular cancer [34]. The role of hypoxia-responsive circRNAs in CRC metabolic reprogramming was unknown. The proliferation of cancer cells increases lipogenesis, and the activation of SREBPs plays a central role in this process. Generally, SREBPs are inhibited by a complex composed of INSIG proteins, SCAP and sterols in the ER [21, 35]. Sterol levels and oncogenic signaling regulate the interaction between INSIG proteins and SCAP- SREBPs complex which is critical for the dissociation of this complex from the ER and the activation of SREBPs [17, 36, 37]. In this study, this protein interaction is regulated by an epigenetic regulation mechanism. Hypoxia-induced circINSIG1 encoded circINSIG1-121 to promote K48-linked ubiquitination of INSIG1 at lysine 156 and 158 by recruiting CUL5-ASB6 complex, leading to the release of SCAP-SREBP2 complex from the ER.

INSIG1 is an ER membrane protein that promotes retention of SREBPs in ER, thereby preventing the proteolytic generation of the transcriptionally active nuclear forms of SREBPs (nSREBPs) and limiting the transcription of SREBPs target genes [38]. Actually, INSIG1 itself is a target of nSREBPs, and its mRNA level rises and falls coordinately with nSREBPs levels [39, 40]. INSIG1 mRNA is expressed at high levels when nuclear SREBP levels are elevated by sterol deprivation. Due to the dependence on SREBPs, INSIG1 mRNA levels decline drastically when cells are overloaded with sterols [41]. Therefore, it is reasonable to speculate that the decrease of INSIG1 protein levels will lead to elevated nSREBPs levels, and then upregulate the expression of downstream target genes including INSIG1. In fact, our study found that overexpression of circINSIG1-121 reduced the protein levels of INSIG1, whereas increased the RNA levels of INSIG1. Moreover, circINSIG1-121 recruited a ubiquitin E3 ligase complex, CUL5-ASB6 complex, to promote the ubiquitination of INSIG1 and mediate its degradation, suggesting that posttranscriptional mechanisms may play a vital role in the inconsistency between INSIG1 protein levels and RNA levels. However, there was no significant difference in INSIG1 protein levels between paired normal adjacent tissues and CRC tissues, although circINSIG1-121 was upregulated in CRC tissues. This might be a result of an increase in INSIG1 transcription owing to SREBP activation, which compensates for the degradation of INSIG1 in a feedback manner. We think that the complex feedback between INSIG1 and SREBPs and the posttranscriptional regulation of INSIG1 may jointly maintain the relatively stable protein level of INSIG1, which warrants further exploration.

The components in the tumor hypoxic microenvironment are associated with poor prognosis of patients [13]. Hypoxic stress favors tumor progression by inducing immunosuppression. In hypoxic zones, HIF-1 directly regulates the function and differentiation of tumor-derived MDSCs. Further evidence supports that HIF-1 is a major regulator of PD-L1 expression [42]. The originally elevated immunosuppressive capacity of tumor-derived MDSCs under hypoxia was found to be abrogated following PD-L1 blockade [43]. Therefore, a better understanding of the pathways in the hypoxic environment during tumor progression is important and may contribute to breakthroughs in cancer therapy. However, in this study, we only focused on the role and mechanism of hypoxia-induced circINSIG1 in CRC cells. It would be interesting to further investigate the role of circINSIG1 in the tumor microenvironment.

## Conclusions

Our findings reveal that hypoxia induces the upregulation of circINSIG1, and the biogenesis of circINSIG1 is regulated by EIF4A3. Moreover, circINSIG1 encodes a novel protein circINSIG1-121, which recruits CUL5-ASB6 complex to promote K48-linked ubiquitination of the critical cholesterol metabolism regulator INSIG1 at lysine 156 and 158, thereby facilitating cholesterol biosynthesis and CRC progression (Fig. 7H). Notably, circINSIG1 presents an epigenetic mechanism that provides new insights into the crosstalk between hypoxia and cholesterol metabolism, and a promising therapeutic target for the treatment of CRC.

## Electronic supplementary material

Below is the link to the electronic supplementary material.

Supplementary Material 1

Supplementary Material 2

Supplementary Material 3

Supplementary Material 4

## Data Availability

The RNA sequencing data used in the study (GSE163868) are available in a public repository from NCBI (https://www.ncbi.nlm.nih.gov/geo/query/ acc. cgi? acc = GSE1 63,868). The mass spectrometry data for circINSIG1-121 and circINSIG1-121 interacting proteins identification have been deposited to the ProteomeXchange Consortium via the PRIDE partner repository with the dataset identifier PXD037292 and PXD037028, respectively. Other data supporting the findings of this study are available from the corresponding authors.

## List of Abbreviations

CRC: Colorectal cancer
circRNA: Circular RNA
HIF-1α: Hypoxia-inducible factor 1α
qRT-PCR: Quantitative real-time PCR
cDNA: Complementary DNA
ER: Endoplasmic reticulum
gDNA: Genomic DNA
FISH: Fluorescence in situ hybridization
ORF: Open reading frame
IHC: Immunohistochemistry
PDOs: Patient-derived organoids
shRNAs: Short hairpin RNAs
IP: Immunoprecipitation
3-MA: 3-methyladenine
SREBPs: Sterol regulatory element–binding proteins
SCAP: SREBP-cleavage-activating protein
CUL5: Cullin5
RBPs: RNA-binding proteins
RIP: RNA immunoprecipitation
PDX: Patient-derived xenograft
