# Supplementary material for: A novel protein encoded by circINSIG1 reprograms cholesterol metabolism by promoting the ubiquitin-dependent degradation of INSIG1 in colorectal cancer
Source: Mol Cancer. 2023 Apr 22;22:72. doi: 10.1186/s12943-023-01773-3 (PMC10122405; doi:10.1186/s12943-023-01773-3)
Supplement: Supplementary file 1 — Supplementary Material 1 [file 12943_2023_1773_MOESM1_ESM.pdf]

Title: A novel protein encoded by circINSIG1 reprograms cholesterol metabolism by promoting the ubiquitin-dependent degradation of INSIG1 in colorectal cancer

Authors: Li Xiong[1, 2], Hua-shan Liu[1, 2], Chi Zhou[3, 4], Xin Yang[1, 2], Liang Huang[1, 2], Hai-qing Jie[1, 2], Zi-wei Zeng[1, 2], Xiao-bin Zheng[1, 2], Wen-xin Li[1, 2], Zhan-zhen Liu[1, 2], Liang Kang[1, 2] and Zhen-xing Liang[1, 2]

From: [1]Department of Colorectal Surgery, The Sixth Affiliated Hospital, Sun Yat-sen University, Guangzhou, Guangdong, China.

[2]Guangdong Provincial Key Laboratory of Colorectal and Pelvic Floor Diseases, The Sixth Affiliated Hospital, Sun Yat-sen University, Guangzhou, Guangdong, China.

[3]State Key Laboratory of Oncology in South China, Collaborative Innovation Center for Cancer Medicine, Sun Yat-sen University Cancer Center, Guangzhou, China

[4]Department of Colorectal Surgery, Sun Yat-sen University Cancer Center, Guangzhou, China

Authorship: Li Xiong, Hua-shan Liu and Chi Zhou contributed equally to this study.

Correspondence authors:

Zhen-xing Liang and Liang Kang

The Sixth Affiliated Hospital, Sun Yat-sen University

26 Yuancun Erheng Rd, Guangzhou, Guangdong, China, 510655

Email: liangzhx5@mail2.sysu.edu.cn; kangl@mail.sysu.edu.cn.

Conflict of interest: The authors have declared that no conflict of interest exists.

Supplemental Figures

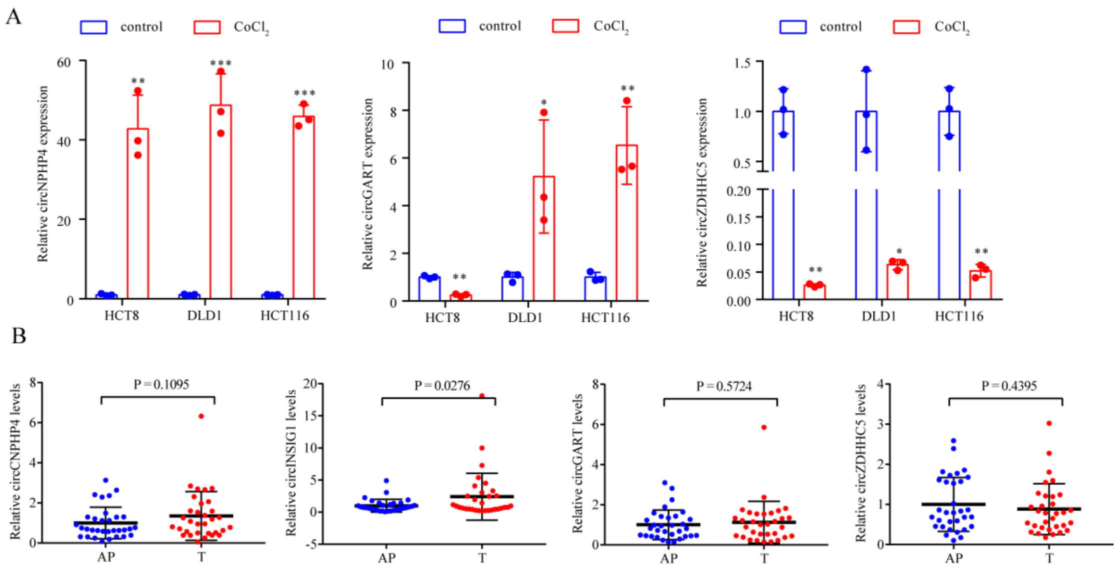

Figure S1. Related to Figure 1. A qRT-PCR analysis of circNPHP4, circGART and circZDHHC5 expression in CoCl2 induced cellular pseudo-hypoxia models. **B** qRT-PCR analysis of circNPHP4, circINSIG1, circGART and circZDHHC5 expression in 32 paired CRC samples and normal **a**djacent tissues.

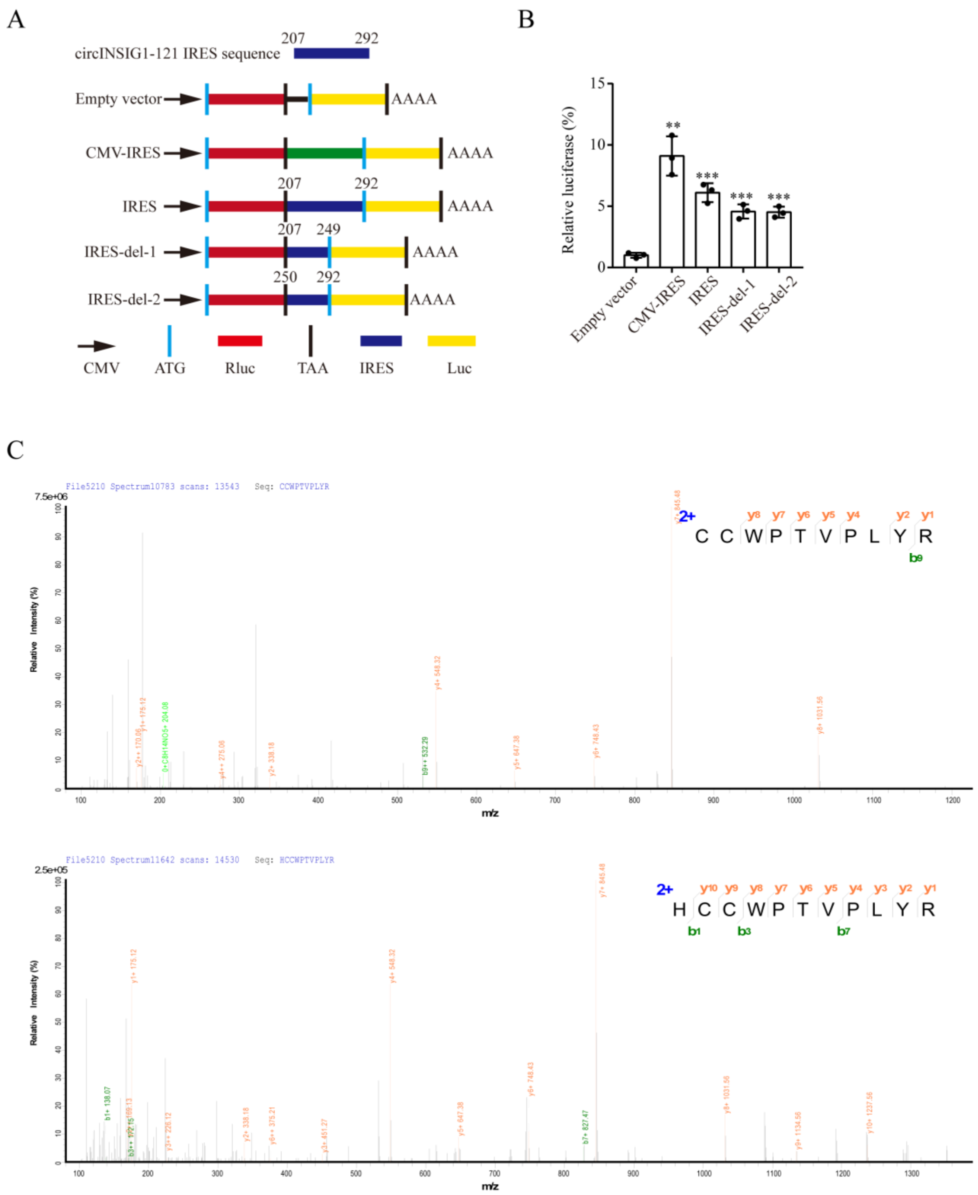

Figure S2. Related to Figure 2. A IRES sequences in circINSIG1 or its different truncations were cloned between Rluc and Luc reporter genes with independent start and stop codons. **B** The relative luciferase activity of Luc/Rluc transfected with indicated vectors was tested. **C** The identified circINSIG1-121 unique peptide sequences. Values are represented as mean ± SD. **$p < 0.01$, ***$p < 0.001$, by one-way ANOVA.

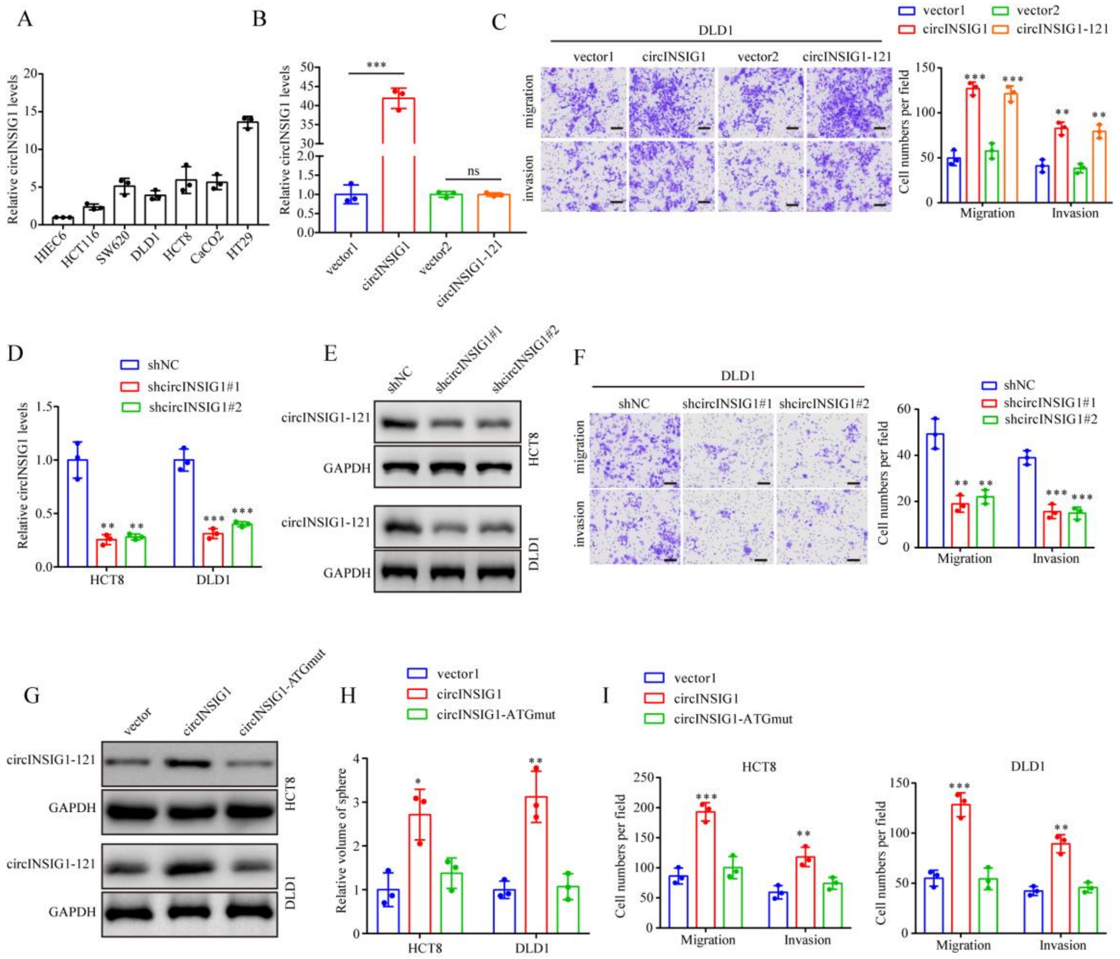

Figure S3. Related to Figure 3. A qRT-PCR analysis of circINSIG1 expression in CRC cell lines. **B** qRT-PCR analysis of circINSIG1 expression in DLD1 cells with circINSIG1 or circINSIG1-121 overexpression. **C** Migration and invasion assays of DLD1 cells with circINSIG1 or circINSIG1-121 overexpression, n = 3. Scale bar = 100 μm. **D** qRT-PCR analysis of circINSIG1 expression in HCT8 and DLD1 cells with circINSIG1 knockdown. **E** Western blot analysis of circINSIG1-121 expression in HCT8 and DLD1 cells with circINSIG1 knockdown. **F** Migration and invasion assays of DLD1 cells with circINSIG1 knockdown, n = 3. Scale bar = 100 μm. **G** Western blot analysis of circINSIG1-121 expression in HCT8 and DLD1 cells with circINSIG1 or circINSIG1-ATGmut vector transfection. **H** Sphere formation assays of circINSIG1 or circINSIG1-ATGmut transfected HCT8 and DLD1 cells, n = 3. **I** Migration and invasion assays of HCT8 and DLD1 cells with circINSIG1 or circINSIG1-ATGmut vector transfection, n = 3. Values are represented as mean ± SD. **p < 0.01, ***p < 0.001; ns, no significance, by one-way ANOVA.

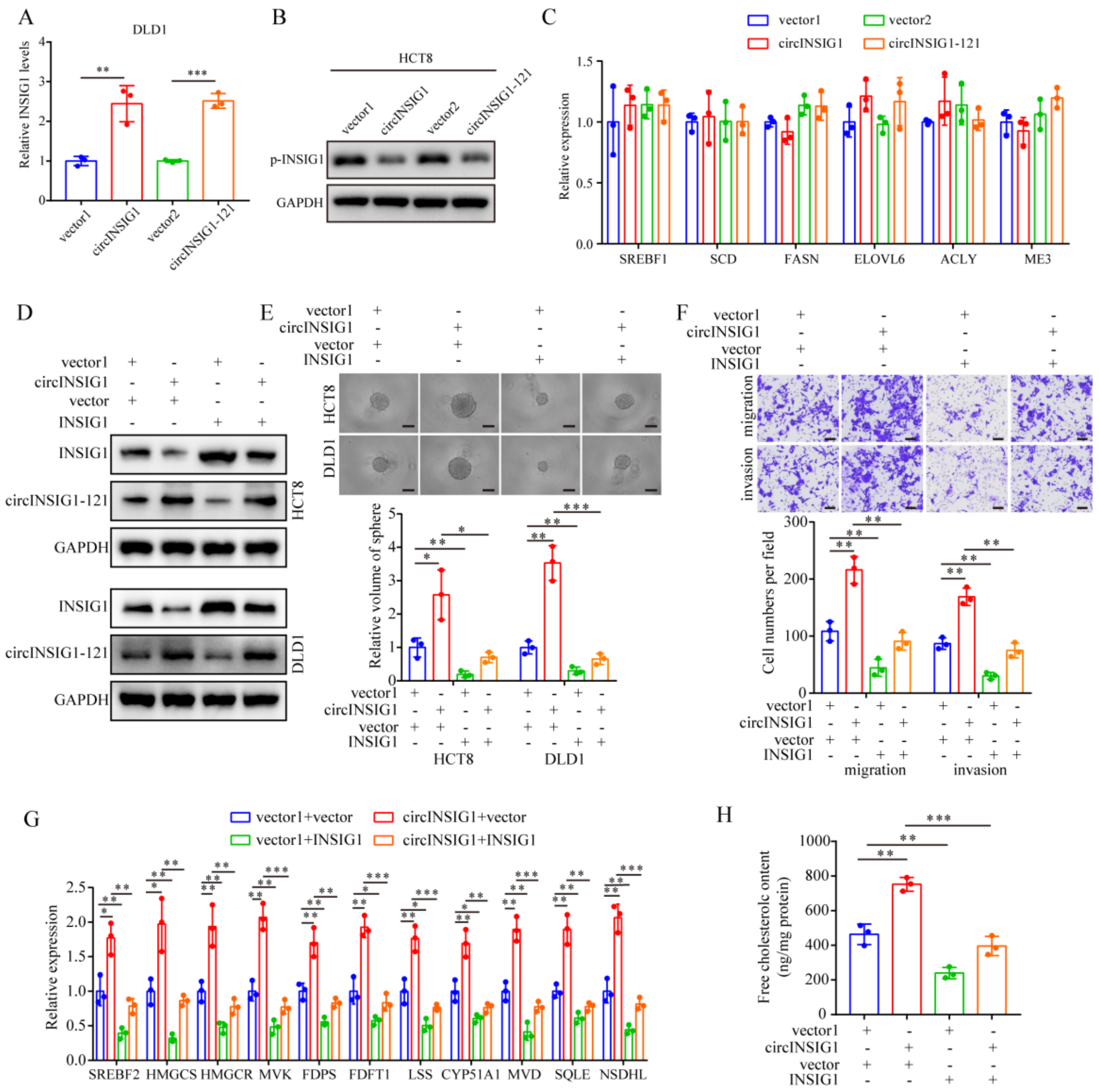

Figure S4. Related to Figure 4. A qRT-PCR analysis of INSIG1 expression in DLD1 cells with circINSIG1 or circINSIG1-121 overexpression. **B** Western blot analysis of phosphorylation of INSIG1 expression in HCT8 cells with circINSIG1 or circINSIG1-121 overexpression. **C** qRT-PCR analysis of SREBP1 target genes in HCT8 cells with circINSIG1 or circINSIG1-121 overexpression. **D-H** HCT8 and DLD1 cells with circINSIG1 overexpression were transfected with INSIG1 plasmid for rescue assays. Western blot analyzed the protein levels of circINSIG1-121 and INSIG1 (**D**). Sphere formation assays evaluated the proliferation of HCT8 and DLD1 cells, n = 3. Scale bar = 200 μm (**E**). Migration and invasion assays of HCT8 cells, n = 3. Scale bar = 100 μm (**F**). qRT-PCR analysis of SREBP2 target genes in HCT8 cells (**G**). Free cholesterol content in HCT8 cells was detected (**H**). Values are represented as mean ± SD. **p < 0.01, ***p < 0.001, by one-way ANOVA.

A

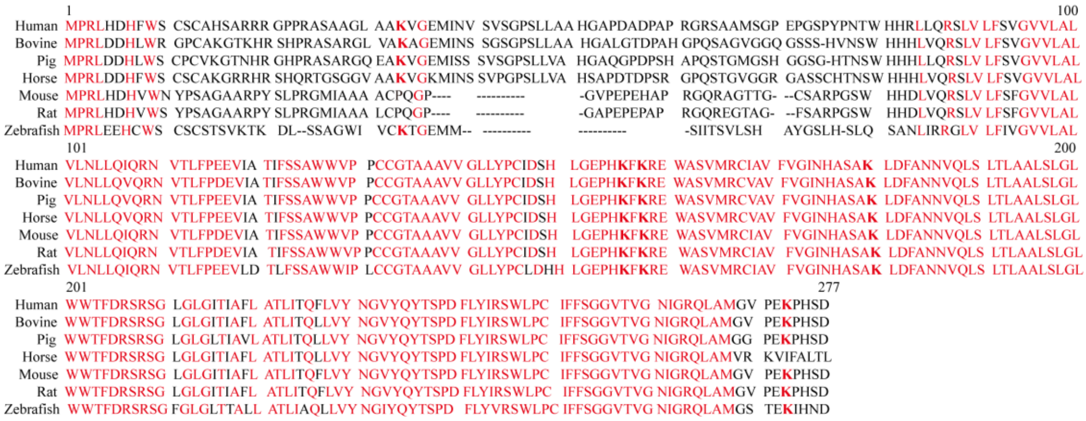

B

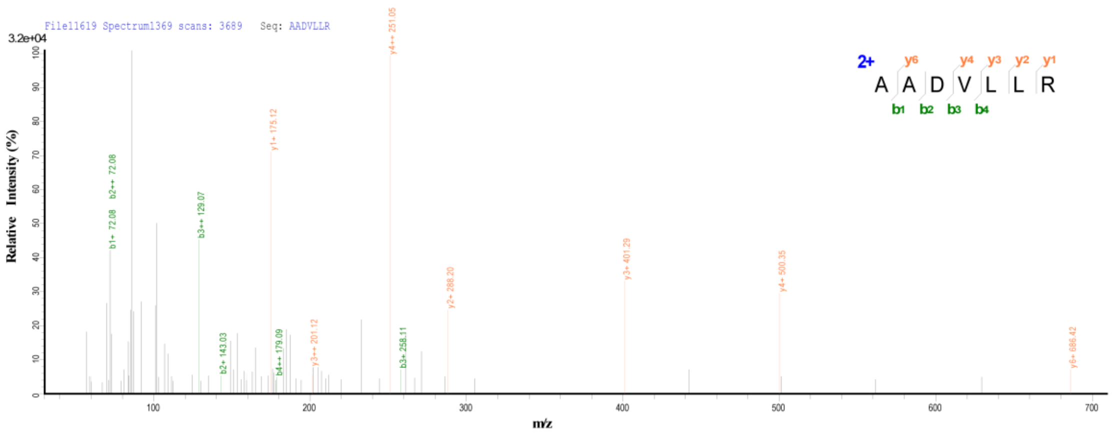

Figure S5. Related to Figure 5. A Alignment of INSIG1 amino acid sequences. Highlighted amino acids indicate conserved lysine (K) of INSIG1. **B** The identified ASB6 unique peptide sequences.

A

circRNA	Tag Name	% Identity	Alignment Length	Mismatches	Gap Openings	Tag Start	Tag End	circRNA Start	circRNA ENd	Upstream/Downstream
hsa_circ_0133744	HHLE1_313256_eIF4AIII_rep1_313256_12_57	100.00	57	0	0	1	57	+374	+430	Downstream
hsa_circ_0133744	HHLE2_1072925_eIF4AIII_rep2_1072925_3_40	100.00	40	0	0	1	40	-679	-640	Upstream
hsa_circ_0133744	HHLE2_1072926_eIF4AIII_rep2_1072926_3_67	100.00	67	0	0	1	67	-493	-427	Upstream
hsa_circ_0133744	HHLE2_1072927_eIF4AIII_rep2_1072927_1_48	100.00	48	0	0	1	48	-419	-372	Upstream
hsa_circ_0133744	HHLE2_1072928_eIF4AIII_rep2_1072928_1_50	100.00	50	0	0	1	50	-344	-295	Upstream
hsa_circ_0133744	HHLE2_1072933_eIF4AIII_rep2_1072933_3_40	100.00	40	0	0	1	40	+261	+300	Downstream
hsa_circ_0133744	HHLE2_1072934_eIF4AIII_rep2_1072934_21_101	100.00	101	0	0	1	101	+330	+430	Downstream
hsa_circ_0133744	HHLE2_1072935_eIF4AIII_rep2_1072935_1_43	100.00	43	0	0	1	43	+435	+477	Downstream
hsa_circ_0133744	HHLE2_1072936_eIF4AIII_rep2_1072936_1_41	100.00	41	0	0	1	41	+621	+661	Downstream
hsa_circ_0133744	HHLE2_1072937_eIF4AIII_rep2_1072937_5_72	100.00	72	0	0	1	72	+721	+792	Downstream
hsa_circ_0133744	HHLE2_1072938_eIF4AIII_rep2_1072938_1_40	100.00	40	0	0	1	40	+936	+975	Downstream

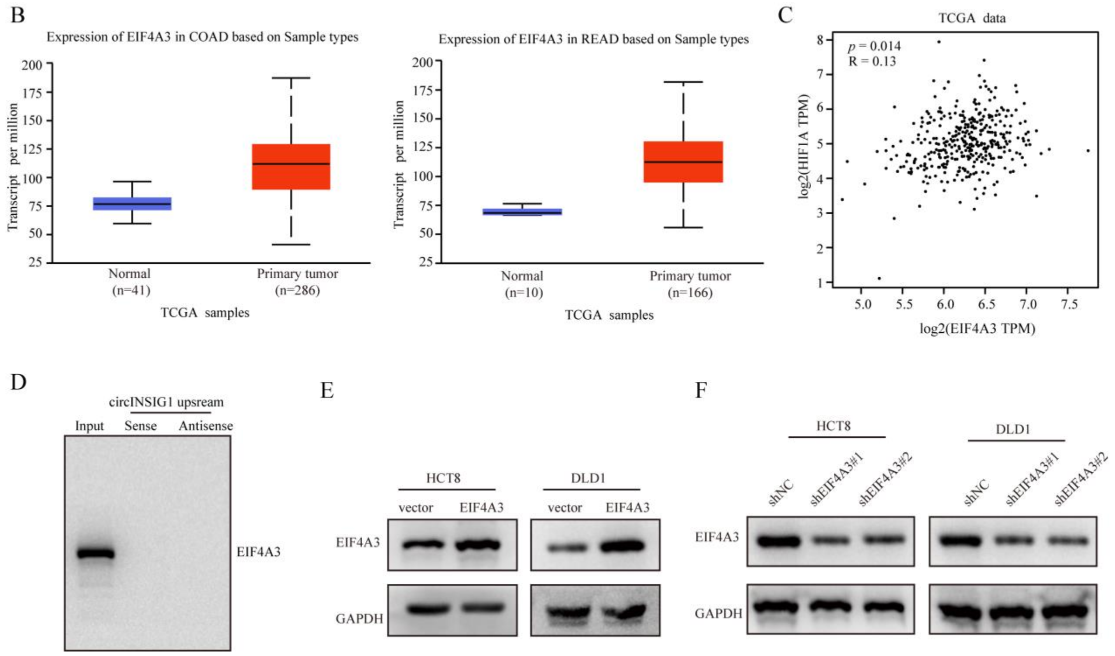

Figure S6. Related to Figure 6. A The EIF4A3 binding sites on the upstream and downstream region of INSIG1 mRNA transcript predicted by the Circular RNA Interactome. **B** The expression of EIF4A3 in CRC in the TCGA database. **C** The correlation between EIF4A3 and HIF-1α expression in the TCGA database. **D** Western blot analysis of RNA pull-down assays with EIF4A3 antibody. **E** Western blot analysis of EIF4A3 expression in HCT8 and DLD1 cells with EIF4A3 overexpression. **F** Western blot analysis of EIF4A3 expression in HCT8 and DLD1 cells with EIF4A3 knockdown.

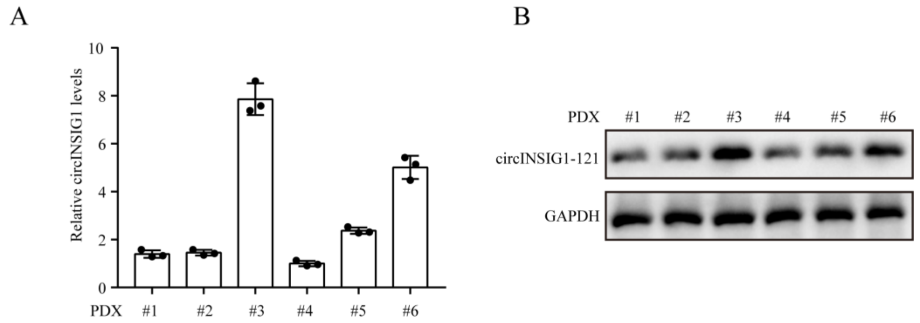

Figure S7. Related to Figure 7. A qRT-PCR analysis of circINSIG1 expression in 6 PDX tumors. **B** Western blot analysis of circINSIG1-121 expression in 6 PDX tumors.

Supplemental Tables

Table S1. Correlation between circINSIG1 expression and clinicopathological characteristics of CRC patients

Characteristics	Frequency	circINSIG1 expression level		
		Low	High	*p*-value[a]
Gender				0.614
Female	101	74	27	
Male	126	96	30	
Age				0.669
≤59	102	75	27	
>59	125	95	30	
T stage				0.005
T1+T2	40	37	3	
T3+T4	187	133	54	
N stage				< 0.001
N0	115	105	10	
N1+N2	112	65	47	
M stage				< 0.001
M0	202	164	38	
M1	25	6	19	
Clinical stage				< 0.001
I+II	112	103	9	
III+IV	115	67	48	

[a] Chi-square test

Table S2. The primers for qRT-PCR

Gene	Forward primer	Reverse primer
18S	CGGCTACCACATCCAAGGAA	GCTGGAATTACCGCGGCT
circINSIG1	GTTCCAGAAGTGGCCTTGGG	GGCGTGGTTAATGCCAACAAA
circNPHP4	ACCTTCTGGTGTCTGGTCTG	GGGAGGGACAAGCACGTTTT
circGART	GAATCCCAACCGCACAATGG	TCTGTCTGTAAAGCAGAAATTCCA
circZDHHC5	GCGTTACCAGTTGAGTCGTG	AACGGAAGCTCTCTGGTTCC
INSIG1	CTCTCGGCCAGGAAGCG	CAGCTCCAGAAGTGGTCGTG
SREBF2	GCCAGGAAGCCCTCTATTGG	CAGAAGAATCCGTGAGCGGT
HMGCS	GGACTGTCCTTTCGTGGCTC	GCCAGCAAGCTTCTGCATTC
HMGCR	AGTGAGATCTGGAGGATCCAAG	TGTCCCCACTATGACTTCCCA
MVK	CTCTGGGTTGTGGGAGTTGG	GGAGCAGACACCAGTAGGAC
FDPS	ACGATACTGTGAGGCATCGG	GACCCCCACAGATCTCAACC
FDFT1	GGACTCGACAGACTCTAAGGC	CAATAAGTCGCCCACGTGTC
LSS	CCTGTGCCATCTCCTACACG	TTGAAGGCCATGGAACGCAC

CYP51A1	TTTCCGACGGAGTGAATGGC	GCAGCATGGACAAGAGGTTG
MVD	ATCAAGTACTGGGGCAAGCG	CAAATCCGGTCCTCGGTGAA
SQLE	GCCTGCCTTTCATTGGCTTC	TTCCTTTTCTGCGCCTCCTG
NSDHL	CAGGAGTTTTGGAAGTGAGAGAAA	ACCGATCACTGTGCATCTCTTG
EIF4A3	GGGCATCTACGCTTACGGTT	GAGCAAGCAGCCCCTGAATA
hHPRT	TTCCTTGGTCAGGCAGTATAATCC	AGTCTGGCTTATATCCAACACTTCG

Table S3. The probe sequence for circINSIG1 FISH and ISH

Gene	**Sequence**
circINSIG1	TACAGTAGGCCAACAACAGTGATAGACACCATTATACA

Supplemental Methods

RNA sequencing

Total RNA was extracted from CRC cell lines (HCT8, HCT116 and DLD1) with or without hypoxic treatment. The RNA purity was analyzed on a Bioanalyzer 2200 instrument (Aligent). Then the RNA was treated with RiboMinus Eukaryote Kit (Qiagen, Valencia, CA) to remove ribosomal RNA and a cDNA library was constructed. Finally deep sequencing was performed with an Illumina HiSeq 3000 (Illumina, San Diego, CA). The clean reads were aligned to the reference genome (GRCH37.p13 NCBI). Unmapped reads were collected to identify the circRNAs. Reads that mapped to the circRNA junction were counted for each candidate.

Patients and samples

Eighty-five paired CRC samples and normal adjacent tissues were used to analyze circINSIG1 RNA levels. None of the patients received chemotherapy or radiotherapy before surgery. All the samples were collected from the Sixth Affiliated Hospital of Sun Yat-sen University. All samples were stored at -80 ℃ refrigerator until further use.

RNA extraction and real-time PCR

Total RNA was isolated from cells by TRIzol Reagent (Thermo Fisher Scientific). ReverTra Ace qPCR RT Kit (Toyobo) was used to perform reverse transcription according to the manufacturer’s instructions. The nuclear and cytoplasmic fractions were isolated by NE-PER™ Nuclear and Cytoplasmic Extraction Reagents (Thermo Scientific).The Applied Biosystems 7500 Sequence Detection system was used to carry out quantitative real-time reverse transcription PCR (qRT-PCR) with the SYBR Green PCR Master Mix (Applied Biosystems). We generated standard curves and

applied the $2^{-\triangle\triangle CT}$ method with normalized to 18S rRNA. We next used the gene of human hypoxanthine-guanine-phosphoribosyltransferase (hHPRT) to quantify cancer metastasis in mouse livers. All the gene-specific primers were obtained from Invitrogen and the oligonucleotide sequences are listed in Table S2.

RNase R treatment

RNase R (Epicentre Technologies, Madison, WI, USA) was used to assess the stability of circRNA. Total RNA (2 μg) was mixed with 0.6ul 10 × RNase R Reaction Buffer and 0.2 μl RNase R or DEPC-treated water (control group). The samples were then incubated at 37 ℃ for 15 min. The expression levels of circINSIG1 and linear INSIG1 were detected by qRT-PCR.

Actinomycin D assay

For the half-life of circRNA assessment, the gene transcription was blocked by adding 2mg/mL Actinomycin D (Sigma-Aldrich, St. Louis, MO, USA) to the cell culture medium. DMSO was used as a negative control. Cells were harvested at 0, 4, 8, 12, 24h and the stability of circINSIG1 and linear INSIG1 was analyzed by qRT-PCR.

RNA fluorescence in situ hybridization (FISH) assay

According to the manufacturer’s instructions, the FISH kit (Ribo Bio, Guangzhou, China) was utilized to perform FISH in cells and the results are visualized by confocal microscopy laser-scanning microscope (Leica TCS-SP8, Leica Microsystems Inc, Buffalo Grove, IL, USA). CRC cells were fixed with 4% paraformaldehyde at room temperature for 10 min, treated with 0.5% Triton X-100 in phosphate buffer saline (PBS) at 4 ℃ for 5 min and then pre-hybridized by Pre-hybridization Buffer at 37 ℃

for 30 min. Finally the cells were hybridized with 2.5 ul 20uM circINSIG1 FISH probes (GenePharma, Shanghai, China) overnight at 37 ℃. The probe sequences are shown in Table S3.

RNA in situ hybridization (ISH) assay

According to the manufacturer's instructions, the ISH Detector kit (BSTER, Wuhan, China) was used to perform ISH in paraffin-embedded CRC tissues. CRC tissues were fixed with 4% paraformaldehyde at room temperature for 10 min; then they were digested with proteinase K at 37 ℃ for 2 min and pre-hybridized at 37 ℃ for 3 h. The tissues were hybridized with double 5'-3'-digoxin (DIG)-labeled circINSIG1 ISH probes (TSINGKE Biological Technology) overnight at 37 ℃. Finally, tissues were incubated with an anti-digoxin monoclonal antibody conjugated with alkaline phosphatase and then incubated with 3, 3'-diaminobenzidine (DAB).

Plasmids construction and stable transfection

To generate the circINSIG1 overexpression vector, the full-length circINSIG1 cDNA was cloned into pLO-ciR (Geneseed Biotech, Guangzhou, China). For circINSIG1-121, INSIG1, ASB6 and EIF4A3-expressing vectors, the full-length ORF sequences of these genes were respectively subcloned into the pSin-EF2-Sox2-Pur or pcDNA3.1 vector (Addgene, Cambridge MA, USA). The activity of the internal ribosome entry site (IRES) was detected with pCMV-IRES-Renilla Luciferase-IRES-Gateway-Firefly Luciferase (pIRIGF) vector. For construction of shcircINSIG1, shASB6 and shEIF4A3, these shRNAs sequences were cloned into pLKO.1 vectors. All constructs were verified by sequencing. For stable transfection, 293T cells were incubated with the vectors described above, psPAX2 and pMD2G

(Addgene) according to the manufacturer’s instructions.

Cell proliferation assays

Cell proliferation was examined using 3D anchorage-free colony formation and patient-derived organoids (PDOs) growth. For 3D anchorage-free colony formation assay, 5,000 cells were seeded in 200 μL DMEM supplemented with 10% FBS in 96-well ultra-low attachment microplate (7007; Corning, USA). Cell culture media was refreshed every three days, and images of 3D colonies were captured using a phase-contrast microscope (DMI4000B, Leica, Wetzlar, Germany). The volume of 3D colonies was calculated using the formula: Volume = 4/3πR3. For PDOs growth assay, PDOs were separated and cultured in the IntestiCult™ Organoid Growth Medium (Human) (STEMCELL, Catalog #06010) according to the manufacturer's instructions. In brief, CRC tissues were washed with ice-cold PBS and then thoroughly minced into the smallest pieces with sterile scissors. Gentle Cell Dissociation Reagent (GCDR) was used to digest the tissue fragments on a rocking platform set at medium speed (~40 rpm) for 30 minutes at room temperature. After aspirate the supernatant, the crypts were calculated and suspended with matrigel (Corning#356231). Finally, the matrigel-crypt suspension was added to the central wells of a 24-well tissue culture-treated plate and the IntestiCult™ Organoid Growth Medium was added.

Migration and invasion assays

Cell migration and invasion were examined using cell migration and invasion assays. Cell migration assays were performed with 24-well plates with 8-μm pore size chamber inserts (Corning). In general, 5×10^4 cells were seeded in the upper chamber well with the non-coated membrane (Millipore) for migration assays. 1×10^5 cells

were seeded in the upper chamber well with the Matrigel-coated membrane (Millipore) for invasion assays. 200 μl of DMEM without FBS was used to resuspend the cells and was added into the upper chamber well. Next, 800 μl of DMEM with 10% FBS was added into the lower chamber. After incubation at 37 ℃ for 24 h, cells migrating through the membrane were stained with 4% paraformaldehyde for 15 min, and then stained with 0.1% crystal violet for 15 min. An inverted microscope (DMI4000B, Leica, Wetzlar, Germany) was utilized to image the cells and software ImageJ (ImageJ 1.46r, Bethesda, MD, USA) was used to quantify the cells.

Immunohistochemistry (IHC)

Paraffin-embedded tissues were deparaffinized with dimethylbenzene followed by antigen retrieval. The tissues were blocked with normal goat serum at 37 ℃ for 30 min. Next, the tissues were incubated overnight at 4°C with specific primary antibodies against circINSIG1-121 (NovoPro Bioscience, 1:100), INSIG1 (sc-390504, 1:100), and ki67 (ab16667, 1:200). Finally, the tissues were incubated with appropriate secondary antibodies and then incubated with 3, 3'-diaminobenzidine (DAB).

Western blot

Cell and tissue samples were lysed with radio-immunoprecipitation assay buffer (RIPA) with protease and phosphatase inhibitors cocktail (Promega). Proteins were separated by SDS-PAGE and then transferred to polyvinylidene fluoride (PVDF) membranes by the Trans-Blot System (Bio-Rad, CA, USA). The membranes were blocked by milk and then incubated with specific primary antibodies against circINSIG1-121 (NovoPro Bioscience, 1:1000), INSIG1 (sc-390504, 1:1000), Flag

(CST#14793S, 1:1000), ASB6 (21449-1-AP, 1:1000), CUL5 (ab184177, 1:1000), p-INSIG1 (SAB663, 1:1000),SREBP1 (MA5-11685, 1:1000), SREBP2 (ab30682, 1:1000), EIF4A3 (ab180573, 1:1000), GAPDH (CST#5174, 1:1000), Ubiqitin (CST#58395, 1:1000) and HA (CST#3724, 1:1000). Finally membranes were incubated with a specific secondary antibody and visualized by ECL Blotting Detection Reagents. GAPDH served as a control for western blot analysis.

RNA immunoprecipitation (RIP)

The RIP assay was performed using a MagnaRIP RNA-Binding Protein Immunoprecipitation Kit (Millipore, MA, USA) according to the manufacturer's instructions. Briefly, the corresponding cell lysates were incubated with beads coated with 5 μg of control IgG antibody or anti-EIF4A3 antibody (ab180573) with rotation at 4 ℃ overnight. Next, total RNA was extracted for the detection of target genes expression by qRT-PCR.
