# Supplementary material for: A novel protein encoded by circINSIG1 reprograms cholesterol metabolism by promoting the ubiquitin-dependent degradation of INSIG1 in colorectal cancer
Source: Mol Cancer. 2023 Apr 22;22:72. doi: 10.1186/s12943-023-01773-3 (PMC10122405; doi:10.1186/s12943-023-01773-3)

Figure 2C

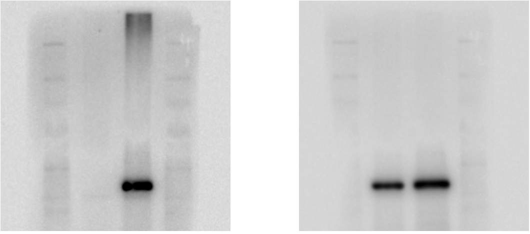

Figure 2G

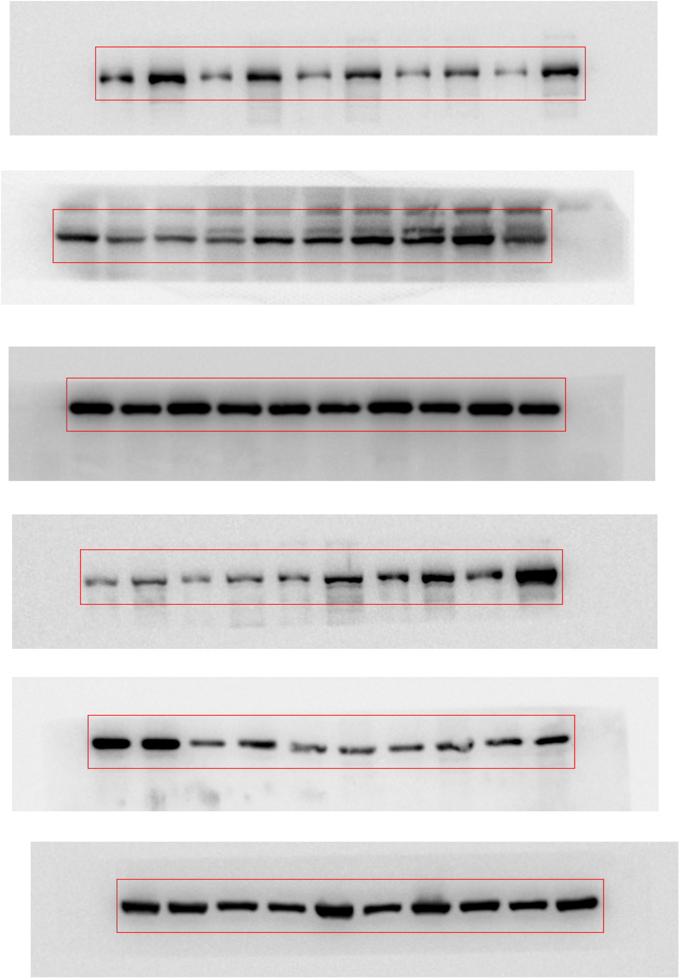

Figure 3B

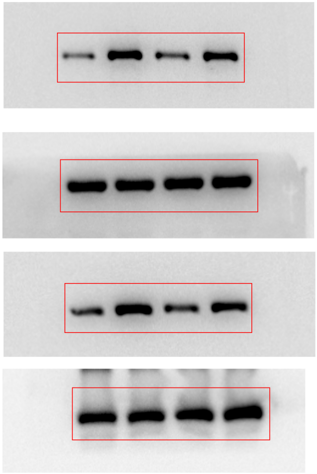

Figure S3E

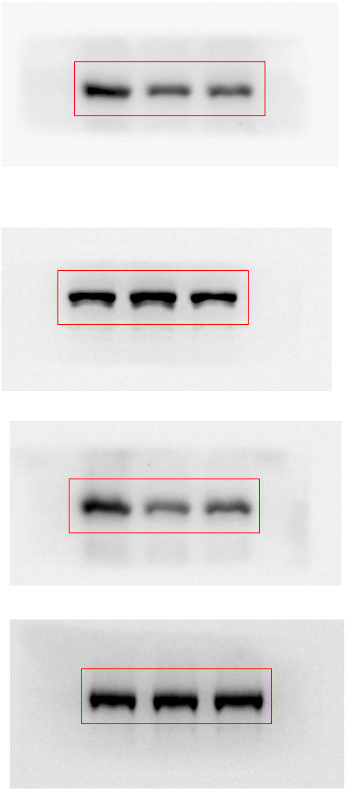

Figure S3G

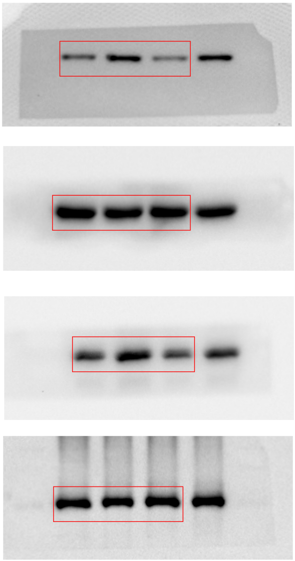

Figure 4B

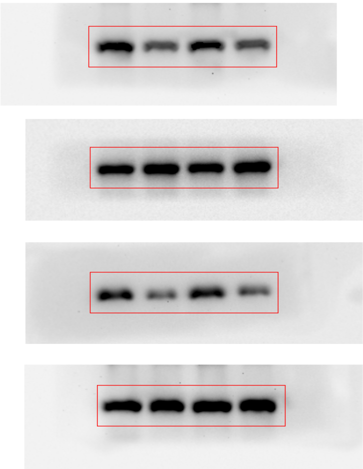

Figure 4D

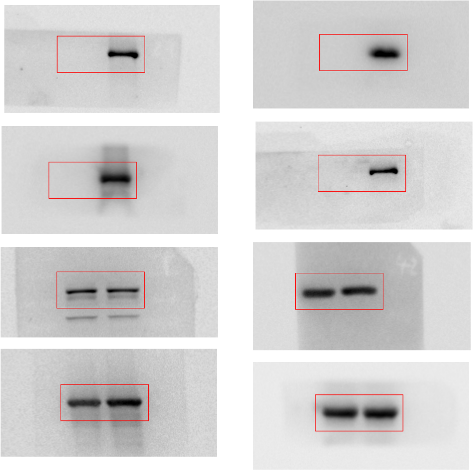

Figure 4E

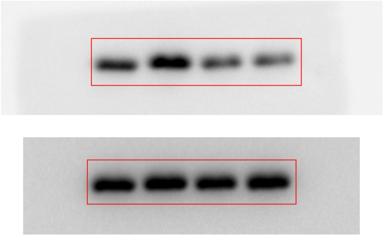

Figure 4F

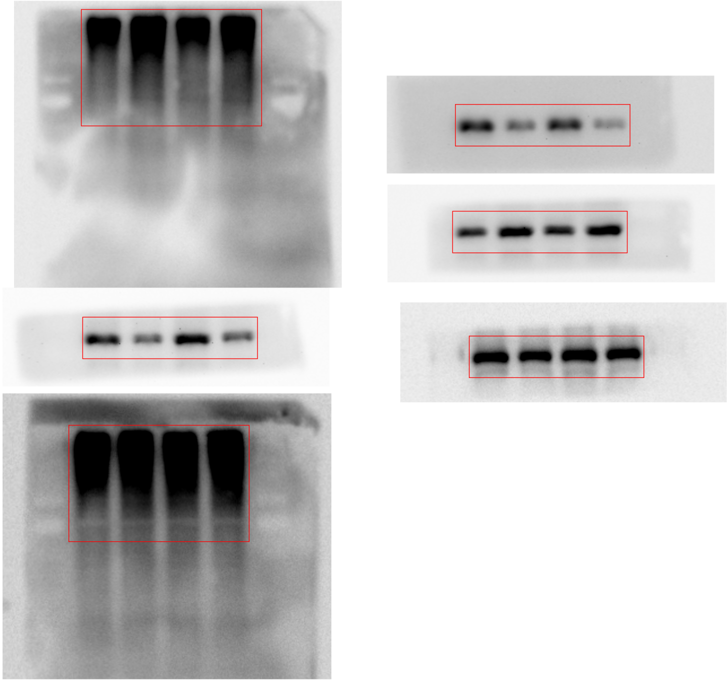

Figure 4G

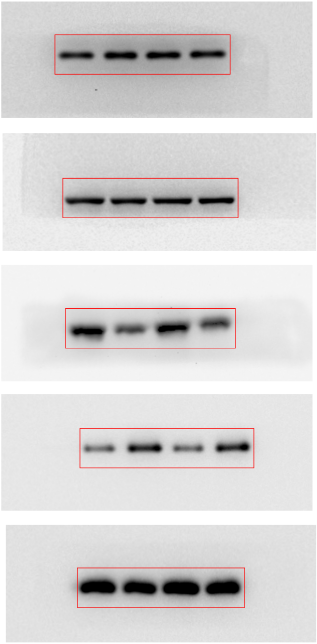

Figure S4B

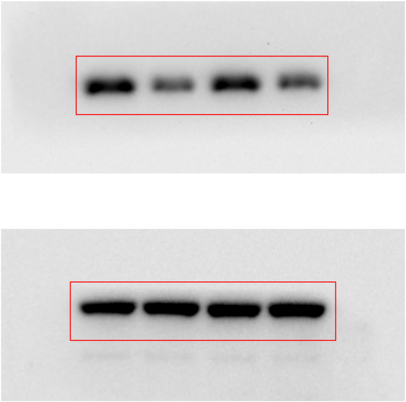

Figure 5A

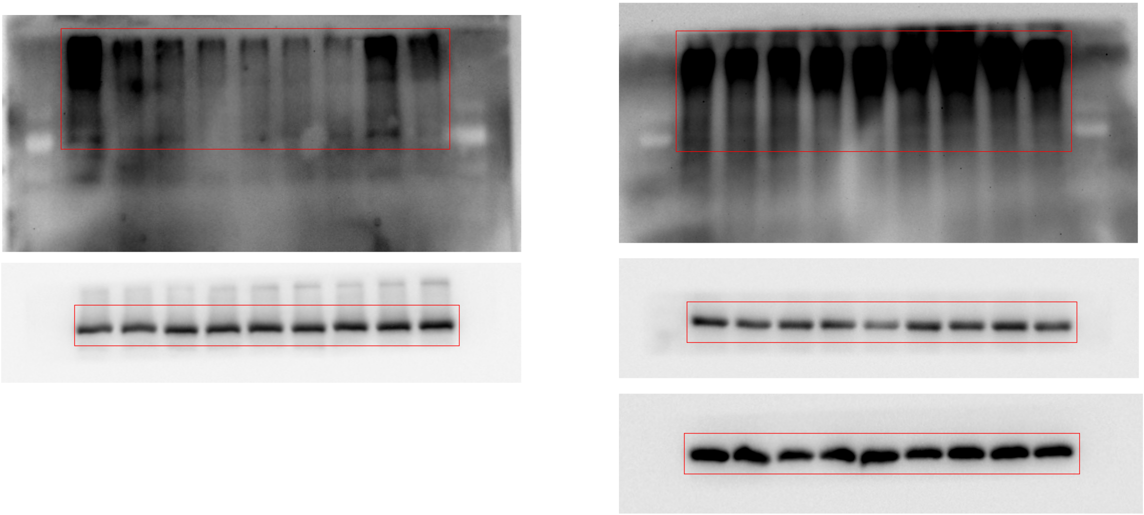

Figure 5B

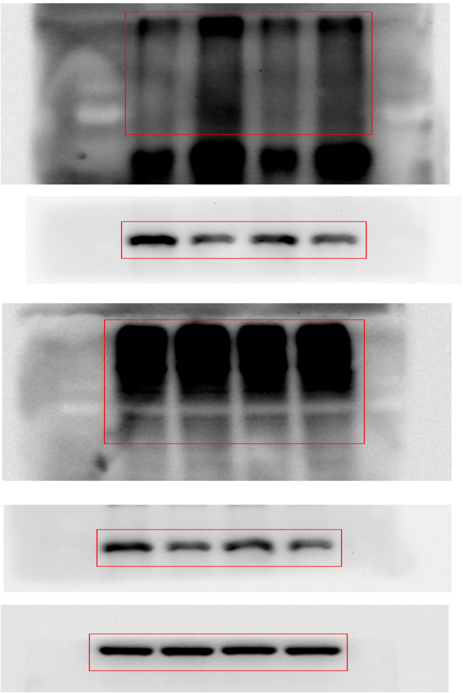

Figure 5C

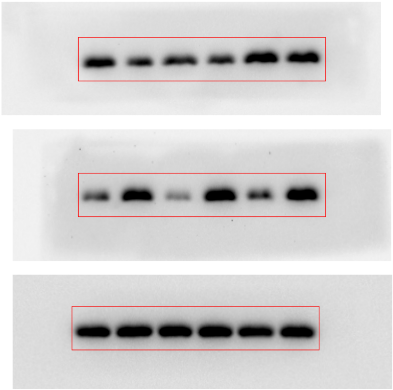

Figure 5D

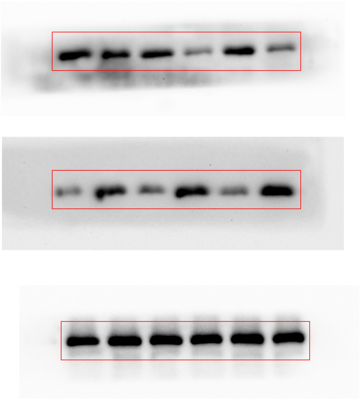

Figure 5E

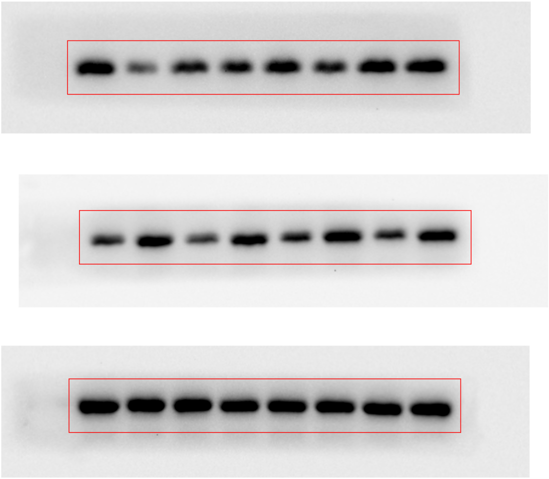

Figure 5F

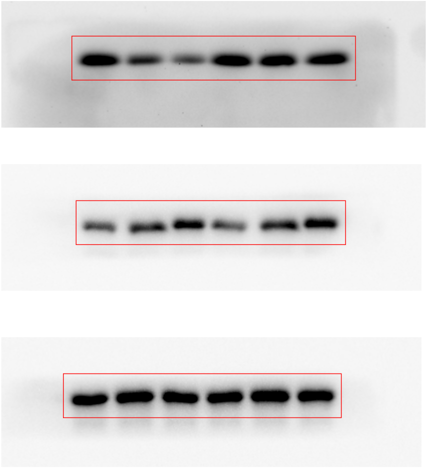

Figure 5G

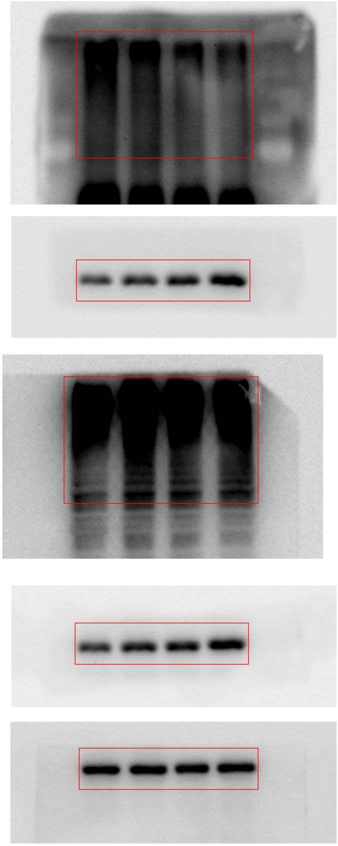

Figure 5H

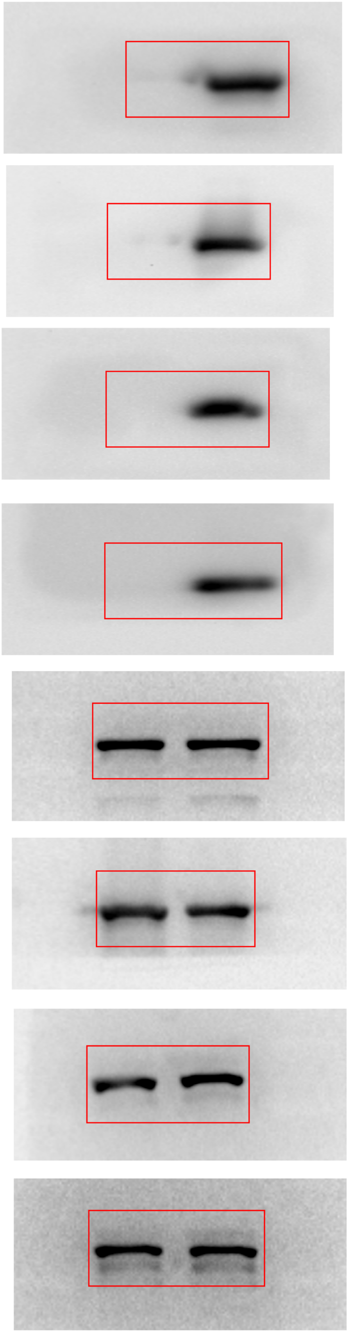

Figure 5I

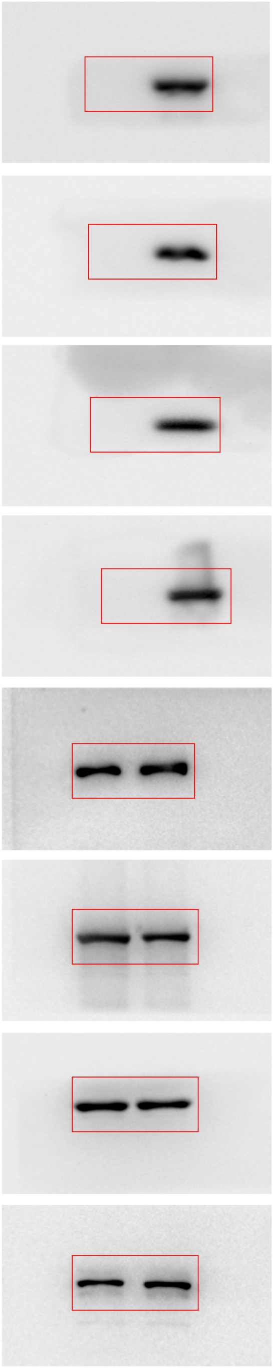

Figure 5J

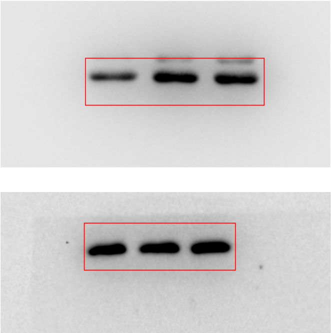

Figure 6B

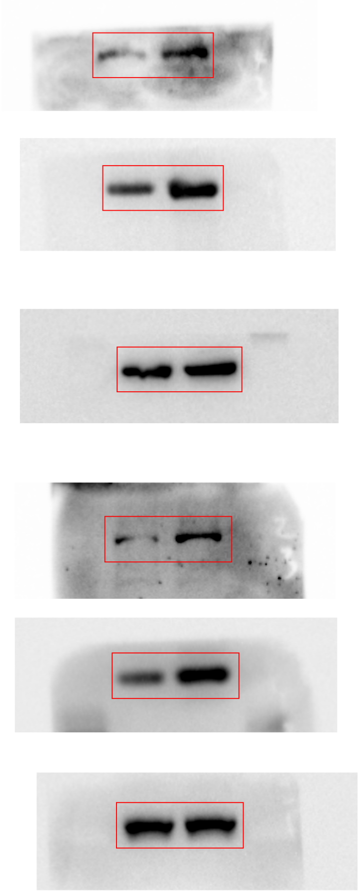

Figure 5K

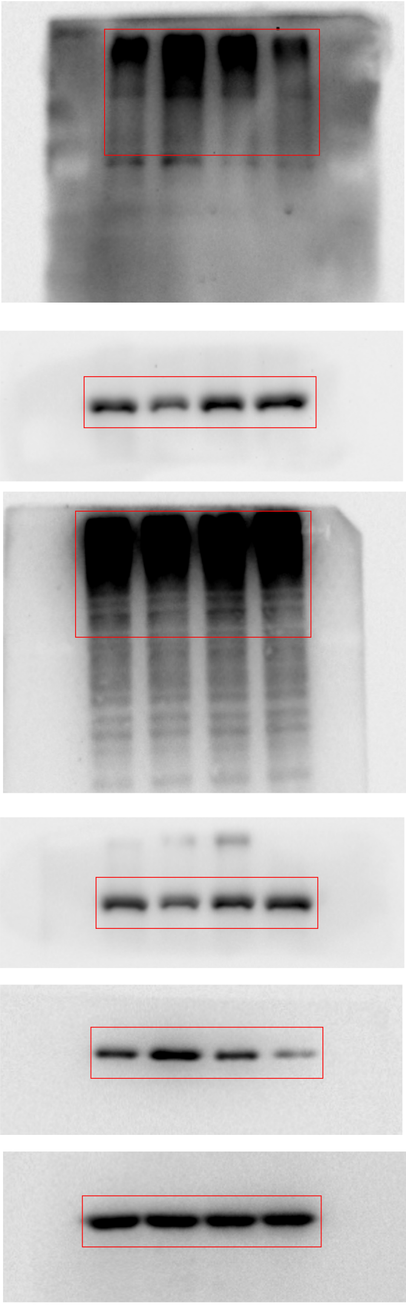

Figure 6D

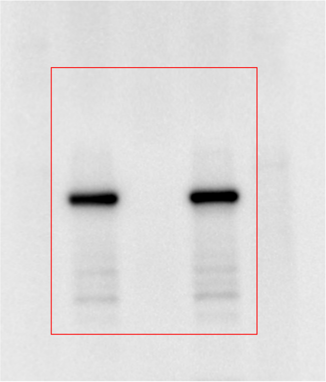

Figure S6D

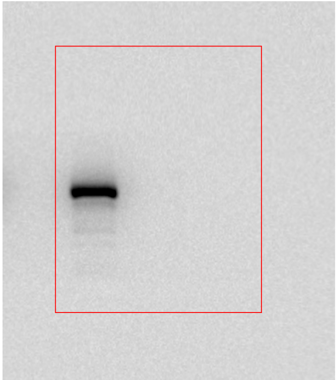

Figure 6K

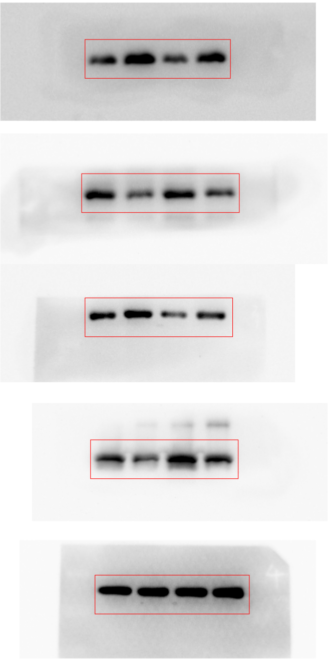

Figure S6E

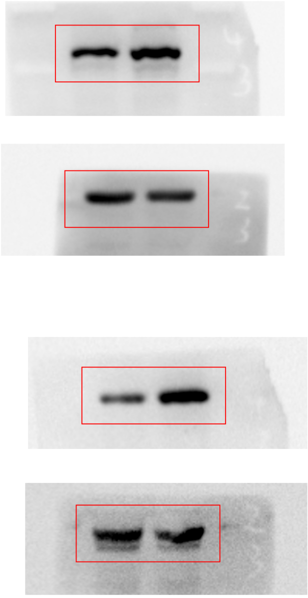

Figure S6F

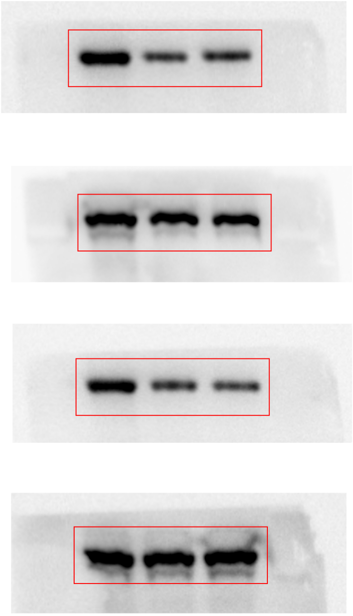

Figure 7D

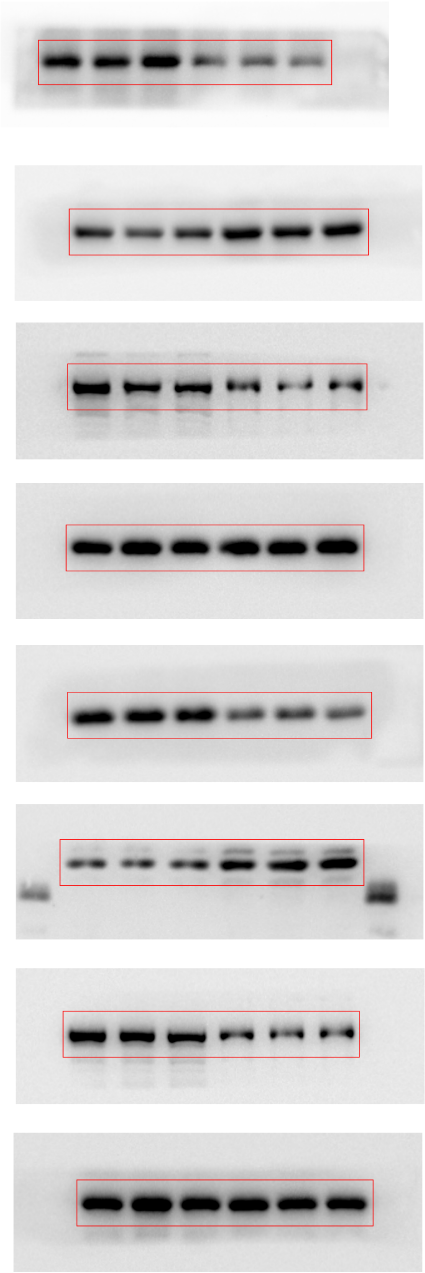

Figure S7B

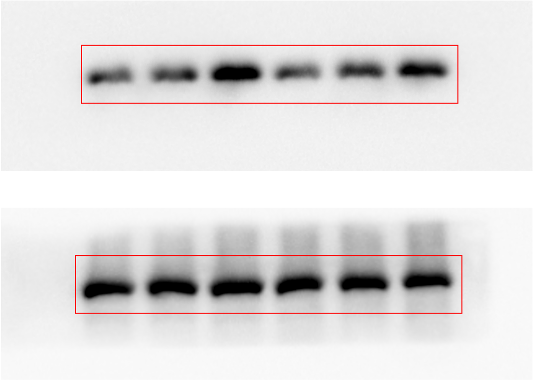

Supplement: Supplementary file 4 — Supplementary Material 4 [file 12943_2023_1773_MOESM4_ESM.pdf]
